# Equity, Diversity, and Inclusion Programs in Health Care Institutions: A Systematic Review and Meta-Analysis

**DOI:** 10.1001/jamanetworkopen.2025.55896

**Published:** 2026-02-04

**Authors:** Deena Fremont, Amos Buh, Claire Hoar-Stephens, Nandini Biyani, Shaafi Mahbub, Ria Singla, Muhammad Zameer, Phalone Mei Nsen, Rachel Kang, Rohan Kiska, Stephen G. Fung, Marco Solmi, Maya Gibb, Mekaylah Scott, Maria Salman, Kathryn Lee, Benjamin Milone, Gamal Wafy, Sarah Syed, Shan Dhaliwal, Ayub Akbari, Pierre A. Brown, Gregory L. Hundemer, Manish M. Sood

**Affiliations:** 1 Ottawa Hospital Research Institute, University of Ottawa, Ottawa, Ontario, Canada; 2 Faculty of Health Sciences, University of Ottawa, Ottawa, Ontario, Canada; 3 Medical Daycare Unit, The Ottawa Hospital, Civic Campus, Ottawa, Ontario, Canada; 4 Faculty of Science, Carleton University, Ottawa, Ontario, Canada; 5 Bruyère Health Research Institute, Ottawa, Ontario, Canada; 6 Department of Psychiatry, University of Ottawa, Ottawa, Ontario, Canada; 7 Department of Mental Health, The Ottawa Hospital, Ottawa, Ontario, Canada; 8 Department of Child and Adolescent Psychiatry, Charité Universitätsmedizin Berlin, Berlin, Germany; 9 Faculty of Medicine, University of Ottawa, Ottawa, Ontario, Canada; 10 Faculty of Science, McMaster University, Hamilton, Ontario, Canada; 11 Division of Nephrology, Department of Medicine, University of Ottawa, Ottawa, Ontario, Canada

## Abstract

**Importance:**

Equity, diversity, and inclusion (EDI) initiatives are politically polarizing and increasingly adopted in the health care setting. Their broader impact across different health care career types, career stages, and various levels of education remains largely unknown.

**Objective:**

To assess EDI programs and their associated outcomes within health care institutions.

**Data Sources:**

A Preferred Reporting Items for Systematic Reviews and Meta-analyses (PRISMA) 2020–compliant systematic review searching PubMed, Scopus, Web of Science, CINAHL, and PsychINFO databases from January 2010 to December 2023.

**Study Selection:**

Two independent reviewers screened studies that assessed EDI programs or policies in health care institutions.

**Data Extraction and Synthesis:**

Programs were categorized based on reported outcomes, including participant satisfaction, increased awareness of EDI-related topics, increases in the proportion of underrepresented minority individuals within medical education or the health care workforce, and overall program impact. Odds ratios (ORs) were pooled using a random-effects model. Analyses followed Preferred Reporting Items for Systematic Reviews and Meta-Analyses reporting guidelines. Analysis was conducted June 2025.

**Main Outcomes and Measures:**

Outcome measures included the proportion of diversity among the workforce, employee and patient satisfaction, and the proportion of employees recruited and retained after program implementation.

**Results:**

In total, 43 studies incorporating more than 15 000 individuals involved in EDI programs were included. Interventions were multifaceted, including 14 career advancement and training programs, 16 diversity representation programs, 11 academia and research support initiatives, and the growth of 2 pipeline programs. Furthermore, interventions demonstrated consistent improvement in EDI initiatives, with perceived benefit in promoting underrepresented minority populations. Findings from the meta-analysis of 2 studies showed that minority representation in competitive medical residencies increased after implementation of 2 EDI interventions (OR, 1.73; 95% CI, 1.21-2.47). Among the 43 studies included in the Joanna Briggs Institute assessment of methodological quality, 7 (16.3%) were rated as high quality, 20 (46.5%) as moderate quality, and 16 (37.2%) as low quality.

**Conclusions and Relevance:**

In this systematic review and meta-analysis of EDI initiatives in health care institutions, programs were associated with an increased workforce diversity. These findings support the continued use of EDI initiatives to promote a more inclusive and equitable health care culture.

## Key Points

**Question:**

What outcomes are associated with equity, diversity, and inclusion (EDI) interventions in health care institutions?

**Findings:**

In this systematic review and meta-analysis of 43 studies involving more than 15 000 individuals, predominantly from the US, a wide range of EDI interventions were successful and perceived as beneficial in increasing diversity in health care. Furthermore, the meta-analysis of 2 studies demonstrated increased minority representation in competitive medical residencies following program implementation.

**Meaning:**

A broad range of EDI initiatives were associated with increased workforce diversity in health care institutions.

## Teaser

This systematic review and meta-analysis assesses equity, diversity, and inclusion initiatives in health care institutions that aimed to promote a more inclusive and equitable health care culture for individuals who beloing to racial and ethnic minority groups.

## Introduction

To address health care disparities in Western medicine and foster trust across patient populations, the promotion of equity, diversity, and inclusion (EDI) in health care institutions has been recommended.^1,2^ Programs include but are not limited to career advancement and training for underrepresented students, diversity representation, and academia and research support initiatives. These programs aim to address systemic barriers and foster culturally informed work environments to ultimately promote equitable access and enhance the cultural competency of health care delivery.^3,4^ Diverse health care workforces appear to improve patient outcomes by enabling culturally sensitive care, promoting health equity, and enhancing the understanding of various population needs.^5^ However, the impact of these programs depends on a variety of factors, such as institutional commitment and accountability to ensure meaningful progress and participant satisfaction.^6^

Despite widespread recognition of the need for EDI initiatives in health care, the current workforce and representation in educational institutions remain largely homogenous.^7^ In 2020, the US health care workforce was composed of approximately 50% White, 20% Asian, 7% Black or African American, and less than 2% Hispanic and Native American (ie, American Indian or Alaska Native) individuals.^7,8^ When assessing representation among medical faculty, this representation decreases, with Black, Hispanic or Latino, and Native American individuals constituting 3.6%, 3.3%, and 0.1% of academic faculty positions, respectively.^9^ These proportions differ noticeably from 2020 Census results of the US general population, which reported 57.8% non-Hispanic White, 18.7% Hispanic or Latino, and 12.1% non-Hispanic Black individuals.^10^ These disparities extend beyond racial representation, encompassing sex representation as well. In the US, only 5.5% of medical school professors and 26% of departmental chairs are female.^9^ Furthermore, recent evidence comparing racial and ethnic representation among recent health care graduates to the current workforce suggests some future incremental improvement in diversity.^11^ However, Black, Hispanic, and Native American individuals were found to remain substantially underrepresented across most health care professionals relative to their proportion in the general population.^11^ This current lack of diversity in the health care workforce poses challenges for caring for diverse patient populations, potentially leading to variable and often detrimental effects on patient outcomes, access to care, and patient trust, as well as workplace experiences and employee retention.^7,9^

The broad impact of EDI programs across different health care career types, career stages, and educational levels currently remains largely unknown.^12^ As such, we conducted a systematic review and meta-analysis to assess the impact (as defined by the original studies) of EDI programs in health care institutions.

## Methods

### Study Design

For this systematic review and meta-analysis, we searched databases from January 2010 to December 2023 following the Preferred Reporting Items for Systematic Reviews and Meta-Analyses (PRISMA) reporting guideline.^13^ The year 2010 was selected as the start of the study period as it aligns with the emergence of formalized EDI policies and interest toward EDI initiatives globally.^14,15,16^ All data to be collected were publicly available and deidentified; institutional review board, ethics committee approval, and informed consent were not needed because data were obtained from existing literature. The review’s protocol was registered with the International Prospective Register of Systematic Reviews (PROSPERO; registration number CRD42024502781) and has been published.^17^

### Inclusion and Exclusion Criteria

This review included studies assessing EDI programs or policies in health care institutions. We included experimental study designs, including randomized clinical trials, cohort and cross-sectional studies, qualitative studies, and preintervention and postintervention studies. Only studies published in English were included due to the team’s language comprehension. Outcome measures included the proportion of diversity among the workforce, employee and patient satisfaction, and the proportion of employees recruited and retained following program implementation.

This review excluded studies if they did not evaluate an EDI program, policy, or related initiative within a health care institution. Furthermore, opinion pieces, commentaries, editorials, conference abstracts, and reviews were excluded from study selection.

### Search Strategy

The full search strategy was created in consultation with a health sciences librarian with expertise in systematic reviews and meta-analysis (eMethods in Supplement 1). We conducted a 3-step strategy to identify relevant studies on EDI programs. First, we conducted an initial search in PubMed, analyzing abstract-level index terms. Initial keyword search terms included *equity*, *diversity*, *inclusion*, *health care facility*, *health care institution*, *hospital*, *health clinic*, *nursing home*, *university*, and *faculty*. Second, the keywords and index terms identified during the initial search were used in our main search across multiple databases. We searched the following databases for articles from January 2010 to December 2023: PubMed, Scopus, Web of Science, CINAHL, and PsycINFO. Finally, we conducted a gray literature search, reviewing reference lists of the studies identified in the previous steps to locate additional relevant studies not captured through the main database search.

### Study Screening

All articles captured in the database searches were imported into the Covidence software for screening. Two reviewers (R. Kang and R. Kiska) independently screened titles and abstracts to identify potentially relevant studies. Any disagreements were resolved with a third reviewer (A.B.). This same procedure was repeated during the full-text screening stage after title and abstract review.

### Data Extraction

Data were extracted using a standardized data extraction tool from the Joanna Briggs Institute (JBI) Manual for Evidence Synthesis.^18^ We extracted the following information: study author and year, study location, description of programs, sample description (including sex and ethnic breakdown if available), and study findings. In the event of missing data, the corresponding study author was contacted. Sex, racial, and ethnic participant information was extracted as defined and reported in the original investigations.

### Data Synthesis

Programs were categorized based on reported outcomes, including participant satisfaction, increased awareness of EDI-related topics, increases in the proportion of underrepresented minority individuals within medical education or the health care workforce, and overall program impact. Due to heterogeneity among interventions and outcome measures, program performance was reported and analyzed according to original study definitions.

### Critical Appraisal

The methodological quality of the included studies was assessed using a standard critical appraisal tool from the JBI for quasi-experimental studies (eMethods in Supplement 1). Based on previous systematic reviews, we assessed methodological quality results in the following categories: studies with scores higher than 70% were considered high quality, studies with scores between 50% and 70% were considered moderate quality, and studies with scores below 50% were considered low quality.^19^

### Statistical Analysis

A meta-analysis was conducted on studies reporting on before-and-after program intervention effects on medical residency enrollment rates for underrepresented minority populations. Odd ratios (ORs) with 95% CIs, considered statistically significant when the interval did not cross the null, were calculated using a random-effects model with inverse variance weighting. Heterogeneity and study variance were assessed using the Paule-Mandel estimator (τ^2^) and *I*^2^ statistics, respectively. Meta-analyses were conducted using the meta package, version 8.1-0, in R, version 4.5.0 (R Project for Statistical Computing). Analysis was conducted June 2025.

## Results

**Figure 1.**
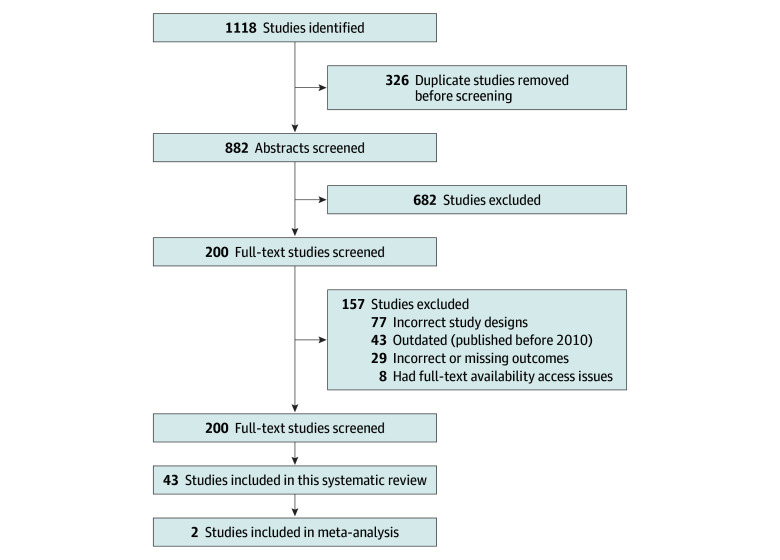
PRISMA Flow Diagram of Selection Process This flow diagram outlines the final 43 studies included in this systematic review^20,21,22,23,24,25,26,27,28,29,30,31,32,33,34,35,36,37,38,39,40,41,42,43,44,45,46,47,48,49,50,51,52,53,54,55,56,57,58,59,60,61,62^ and the 2 studies included in the meta-analysis.^41,56^

A total of 1118 studies were identified after the initial database search (Figure 1). Next, 236 duplicate studies were removed before abstract screening. During abstract screening, another 682 studies were removed. The remaining 200 studies proceeded to full-text review. During this process, 157 studies were further excluded for the following reasons: incorrect or missing outcomes (n = 29), incorrect study design (n = 77), publication date before 2010 (n = 43), and lack of full text availability (n = 8) (eTable 1 in Supplement 1). The remaining 43 studies were included in this systematic review,^20,21,22,23,24,25,26,27,28,29,30,31,32,33,34,35,36,37,38,39,40,41,42,43,44,45,46,47,48,49,50,51,52,53,54,55,56,57,58,59,60,61,62^ and 2 were further used for meta-analysis.^41,56^

### Study Characteristics

All studies were conducted in the US except for a single study from the United Kingdom.^30^ Approximately one-third of studies reported on the sex distribution among the study and program participants, with the studies that did so reporting a majority-female population of program participants.^21,22,25,27,28,31,32,37,38,51,60,62^ Study sample sizes varied greatly, ranging from small-scale mentoring programs (n = 4)^40^ to large, coordinated diversity pipeline initiatives spanning from kindergarten to undergraduate students (n = 91 901).^44^ Whether explicitly stated in their objectives or not, the most frequently reported minority population across studies was Black and African American individuals.^20,21,27,33,37,38,43,51,62^ Several studies also included predominantly Latino^21,35,62^ and Asian^25^ populations. Few studies specified the inclusion of individuals of Middle Eastern,^23^ American Indian,^32,33,34^ or Pacific Islander descent.^33,35^ Detailed characteristics of the studies are summarized in Table 1.

**Table 1.** Characteristics of Included Studies

Source	Country	Sample size^a^	Sample description^a^	
			Sex breakdown	Race and ethnicity breakdown
Mason et al,^20^ 2016	US	118 Medical students from 29 medical schools	Women (41%), men (59%)	Asian (3%), Black (69%), Latino (14%), Native American (5%), White (9%)
Estape et al,^21^ 2018	US	173 Scholars (93 scholars in the Master of Science in Clinical and Translational Research program and 80 scholars in the Master of Science in Clinical Research program)	Master of Science in Clinical and Translational Research program: women (62.4%), men (37.6%); Master of Science in Clinical Research program: women (70%), men (30%)	Master of Science in Clinical and Translational Research program: Latino or Hispanic (100%); Master of Science in Clinical Research program: Asian, non-Hispanic (15%); Latino or Hispanic (2.5%); Black or African American, non-Hispanic (78.8%); White, non-Hispanic (3.8%)
de Dios et al,^22^ 2014	US	14 Mentees and 29 mentors	Mentees: 13 women (92.9%), 1 man (7.1%); mentors: 18 women (62.1%), 11 men (37.9%)	Mentees, self-selected categories: 2 Asian (14.3%), 3 Black (21.4%), 8 White (57.1%), 1 biracial (7.1%); mentors, self-selected categories: 3 Asian (10.3%), 1 Latino (3.4%), 18 White (62.1%), 2 biracial (6.9%), 5 unknown (17.2%)
Inglehart et al,^23^ 2014	US	50 Mentees and 40 mentors	Mentees: 22 women (44%), 28 men (56%); mentors: 30 women (75%), 10 men (25%)	Mentees: 42 Black (84%), 3 Middle Eastern or Indian (6%), 5 White (10%); mentors: 18 Black (45%), 1 Asian (2.5%), 3 Latino (7.5%), 4 Middle Eastern or Indian (10%), 14 White (35%)
Blanchard et al,^24^ 2019	US	1069 Attendees at annual meetings from 2008 to 2018 (junior researchers: postdoctoral fellows and assistant professors [66%], senior researchers: associate and full professors [34%])	Not reported	Not reported
Goldstein et al,^25^ 2014	US	99 Postresidency fellows	50 Women (50.5%), 49 men (49.5%)	42 Asian (42.2%), 25 Black (25.3%), 32 Hispanic (32.3%)
Rice et al,^27^ 2014	US	58 Mentees	Women (62.1%), men (37.9%)	53 Black (91.2%), 3 Hispanic (5.2%), 1 other (1.7%)
Gotian et al,^28^ 2017	US	245 Students	152 Women (62%), 93 men (38%)	209 URM groups (defined as African American, Hispanic American, and Native American) (85.3%)
Aguila et al,^29^ 2010	US	1096 Investigators and students	Not reported	Not reported
Adhikari et al,^30^ 2023	United Kingdom	79 Nurses and midwives from a Black and minority ethnic background and 220 line-managers and 36 mentors	Not reported	Not reported
Dillard et al,^31^ 2018	US	51 Part 1 participants, 24 part 1 and 2 participants	Part 1 participants: 33 women (64.7%), 18 men (35.3%); part 1 and 2 participants: 14 women (58.3%), 10 men (41.7%)	Part 1 participants: 19 Black (37.3%), 27 White (52.9%), 5 other (9.8%); part 1 and 2 participants: 12 Black (50%), 7 White (29.2%), 5 other (20.8%)
Corbie et al,^32^ 2022	US	29 Participants	17 Women (58.6%), 12 men (41.4%)	2 American Indian (6.9%), 4 Asian (13.8%), 7 Black (24.1%), 14 White (48.3%), 2 other (6.9%)
Goldsmith et al,^26^ 2014	US	53 Seventh grade students	Girls (33%), boys (67%)	Asian (10%), Black (33%), Hispanic (19%), Native American (4%), White (35%)
Maton et al,^33^ 2012	US	487 Undergraduate students	Not reported	156 African American (53.4%), 1 American Indian (0.3%), 54 Asian or Pacific Islander (18.5%), 17 Hispanic (5.8%), 64 White (21.9%)^b^
Buchwald et al,^34^ 2011	US	29 American Indian trainees and mentors	Not reported	29 American Indian (100%)
Guerrero et al,^35^ 2015	US	32 Students	Not reported	Asian (29%), Black (16%), Latino (34%), Pacific Islander (6%), Southeast Asian (9%), White (3%), other (3%)
Dossett et al,^36^ 2019	US	Faculty, staff, and residents (total not reported)	Applicant pool: women (55%), men (45%); hires: women (50%), men (50%)	Applicant pool: URM groups (15%); hires: URM groups (33%)
Diefenbeck et al,^37^ 2021	US	29 Freshman nursing students	25 Women (86.2%), 4 men (13.8%)	3 Asian (10.3%), 7 Black (24.1%), 7 Hispanic (24.1%), 4 White (13.8%), 6 other (20.7%)
Gates et al,^38^ 2013	US	15 Participants (5 faculty members [33.3%], 10 residents [66.6%])	11 Women (73.3%), 4 men (26.7%)	8 African American (53.3%), 1 Hispanic (6.7%), 4 Southeast Asian (26.7%), 2 White (13.3%)
Taylor et al,^62^ 2019	US	33 Students	24 Women (72.7%), 9 men (27.3%)	12 African American (36.4%), 5 Asian (15.2%), 12 Hispanic (36.4%), 4 White (12.1%), 5 other (15.2%)
Brown et al,^40^ 2019	US	4 Scholars	Not reported	Not reported
Guevara et al,^61^ 2018	US	Participants: 124 final-round applicants to the faculty development program between 2003 and 2008; scholars: 76 funded participants; non-scholars: 48 unfunded participants)	Not reported	Not reported
Spottswood et al,^41^ 2019	US	777 Applicants, 32 residents	Not reported	Not reported
Germino et al,^42^ 2023	US	35 Radiation oncology residents (mentor-mentee pairs); 31 (88.6%) mentees completed the preprogram survey; 17 (48.6%) mentees completed the postprogram survey	Not reported	Not reported
Youmans et al,^39^ 2020	US	35 URM residents participated as mentors for 50 URM medical students annually; resident mentors participated for an average of 3 to 4 h each year; 20 of 32 eligible resident mentors (63%) completed the survey	Not reported	Not reported
Odedina et al,^43^ 2022	US	40 URM students who completed the program between 2012 and 2019; response rate was 73% with 29 participants	Not reported	Black or African American (89.7%)
Vishwanatha et al,^44^ 2019	US	7531 Kindergarten to grade 12 participants; 762 undergraduate students; 25 postbaccalaureate students (3.3%); 229 predoctoral students (30.1%); 150 postdoctoral fellows and junior faculty (19.7%)	Women (62%), men (38%)	URM groups (83%)
Butler et al,^45^ 2015	US	76 Surgical residents that participated from 2002 to 2009	Not reported	Not reported
Murray et al,^46^ 2016	US	392 Racial and ethnic minority and/or disadvantaged high school students attended the health career clubs; 310 (79%) expressed intent to pursue a health-related or nursing career; 185 nursing students (47.2%) enrolled in retention programs	Not reported	Not reported
Pachter et al,^47^ 2015	US	65 URM students entering careers in academic pediatrics; 65 (100%) completed the program	Not reported	Not reported
Llado-Farrulla et al,^48^ 2021	US	Residents of University of Pennsylvania plastic and reconstructive surgery residency program (during a 9-y period, values on the number of program enrolments were not reported)	Not reported	Not reported
Alli et al,^49^ 2023	US	205 Preclinical medical students across 3 campuses; supported by 60 faculty members and facilitators; 160 students (78%) submitted course evaluations	Not reported	Not reported
Williams et al,^6^ 2020	US	18 Peer mentors in 3 peer mentor development program cohorts	Not reported	Not reported
Metz et al,^51^ 2017	US	525 Students entering the program between 1995 and 2009	Women (69.7%), men (30.3%)^c^	American Indian (<1%), Asian (1.1%), Black (83.2%), Hispanic or Latino/a (10.5%), White (4.2%)
Travers et al,^52^ 2015	US	5 States (Arkansas, California, Michigan, Florida, Texas) implementing laws to assist recruitment of racial and ethnic minority groups in nursing schools	Not reported	Not reported
Flores et al,^53^ 2021	US	10 Scholars from the first 4 cohorts; additional 33 URM young investigators involved in the first 4 conferences	Not reported	Not reported
Eakin et al,^54^ 2022	US	102 Racial and ethnic minority researchers at Michigan Institute for Clinical and Health Research across 5 cohorts; 3 in-person cohorts (57 researchers [55.9%]); 2 remote cohorts (45 researchers [44.1%])	Not reported	Not reported
Zhou et al,^55^ 2021	US	15 URM undergraduate students in 2020	9 Women (60%), 6 men (40%)	1 Asian (6.7%), 11 Black (73%), 3 Hispanic (20%)
Harris et al,^56^ 2012	US	26 Students	Not reported	13 URM groups (listed broadly as African American, Asian American, Egyptian American, and Latino American) (50%)
Greenway et al,^57^ 2021	US	98 Scholars	Not reported	Hispanic or Latino (24%), non-White (72%)
Degazon et al,^58^ 2012	US	87 Nursing students from URM and disadvantaged backgrounds	Not reported	9 Black (10%)
Wides et al,^59^ 2013	US	94 Socioeconomically or educationally disadvantaged students from 1998 to 2006	Not reported	URM groups (37%)
Brimhall et al,^60^ 2018	US	247 Employees across 21 departmental units (average of 10 employees per unit)	142 Women (69%), 64 men (31%)^d^	Self-selected categories: 102 Asian (41%), 10 Black (4%), 35 Hispanic (14%), 51 White (21%), 49 mixed race or other (20%)

Abbreviations: STEM, science, technology, engineering, and mathematics; URM, underrepresented minority.

^a^ Data are counts unless percentages are presented.

^b^ Composition of students in most recent years.

^c^ Age range from 20 to 45 years.

^d^ Age range from 18 to 70 or more years.

### Program Types

The 43 studies included in this review can be grouped under 4 broad strategies: (1) career advancement and training (n = 14),^22,28,30,32,38,39,42,45,47,50,51,56,57,58^ (2) diversity representation (n = 16),^20,23,26,27,35,36,37,41,44,46,48,52,55,59,60,62^ (3) academia and research support initiatives (n = 11),^24,25,29,31,33,34,40,43,53,54,61^ and (4) the growth of pipeline programs (n = 2)^21,49^ (Table 1).

### Program Outcomes

The outcomes of most EDI programs were broadly positive, with high participant satisfaction across health care settings (Table 2).^22,23,31,37,38,42,54,60,62^ In the health care workforce, outcomes varied by profession and career stage. For early career physicians, studies reported increased underrepresented minority representation in competitive residency programs, including plastic and reconstructive surgery (representation increased from 0% to 29% during a 9-year period),^48^ radiology (applications from underrepresented minority individuals increased from 7.5% to 12.6% during a 6-year period),^41^ psychiatry (representation increased from 40% to 50% during a 4-year period),^56^ and orthopedic surgery (higher proportion of underrepresented minority individuals applied compared with national controls [31% vs 3%, respectively] during a 7-year period).^20^ Among 10 nursing and midwifery professionals, programs facilitated career advancement through promotions to senior roles (n = 1),^30^ pursuit or support of further education (n = 4),^30,37,52,58^ enhanced confidence, communication, and leadership skills (n = 2),^30,37^ retention of nursing professionals (n = 2), and the amendment of state legislation to promote minority recruitment (n = 1).^52^ At earlier career stages, EDI initiatives contributed to improved performance on standardized examinations such as the Medical College Admission Test (MCAT),^51^ Dental Admission Test (DAT),^57^ and the National Council Licensure Examination (NCLEX) for registered nurses.^46,58^ Finally, in the dental industry, 7 programs included a variety of strategies to increase representation of minority groups, including financial assistance for reapplications (n = 1),^57^ career readiness training and exposure initiatives (n = 3),^23,38,59^ and pipeline mentoring programs within underrepresented minority communities (n = 3).^23,57,59^

**Table 2.** Study Outcomes Representative Findings

Source	Representative findings	Outcome category
Mason et al,^20^ 2016	Increased URM (defined as Black and Latino) and female representation	Diversity representation
Estape et al,^21^ 2018	Increased URM representation: increased the number of clinical translational researchers from minority and underrepresented populations Program impact: increased the number of total and successful grant applications from Latino or Hispanic scholars and Black or African American scholars	Growth of pipeline programs
de Dios et al,^22^ 2014	Increased awareness: diversity Participant satisfaction: with program and mentor-mentee matching	Career advancement and training
Inglehart et al,^23^ 2014	Increased URM representation: dental education and industry Participant satisfaction: in overall program ratings	Diversity representation
Blanchard et al,^24^ 2019	Program impact: supporting URM populations in research	Academia and research support initiatives
Goldstein et al,^25^ 2014	Program impact: increased URM mental health research Increased URM representation: minority psychiatrists in research careers	Academia and research support initiatives
Rice et al,^27^ 2014	Program impact: increased URM publications, research productivity, and career development	Diversity representation
Gotian et al,^28^ 2017	Increased URM representation: advanced degrees in science and medicine	Career advancement and training
Aguila et al,^29^ 2010	Increased URM representation: in clinical research trials and subsequent publications and grants	Academia and research support initiatives
Adhikari et al,^30^ 2023	Program impact: advancements in URM careers, and improvement in confidence and communication skills Increased awareness: URM students felt better supported by management	Career advancement and training
Dillard et al,^31^ 2018	Program impact: increased quality of life (improvements in depression, autonomy, mental health, functional health literacy, and positive beliefs about research), education, improved mental state of diverse seniors Increased awareness: participants completing both program components were also more active in postprogram advocacy, highlighting the program’s performance in empowering diverse seniors to engage in and promote health research Participant satisfaction: participants rated classes positively	Academia and research support initiatives
Corbie et al,^32^ 2022	Increased awareness: significant growth in organizational, community engagement, and system change competencies Program impact: increased equity diversity inclusion -related knowledge, skills, and self-efficacy among participants	Career advancement and training
Goldsmith et al,^26^ 2014	Program impact: increased understanding of health care careers among young URM students	Diversity representation
Maton et al,^33^ 2012	Increased URM representation: increased successful entrance of URM students into a PhD program in the STEM fields Program impact: increased STEM graduation rates and higher GPAs	Academia and research support initiatives
Buchwald et al,^34^ 2011	Program impact: increased American Indian career development, grant application, and manuscript publications	Academia and research support initiatives
Guerrero et al,^35^ 2015	Increased awareness: child health, maternal health, services and programs for children and families, and cultural competency	Diversity representation
Dossett et al,^36^ 2019	Increased URM representation: new faculty hires	Diversity representation
Diefenbeck et al,^37^ 2021	Program impact: increased retention of URM nursing students, and an increase in academic success and professional development Participant satisfaction: participants were very satisfied with the retention program	Diversity representation
Gates et al,^38^ 2013	Program impact: increased confidence in academic skills and increased career development for URM students Participant satisfaction: uniformly positive program reviews	Career advancement and training
Taylor et al,^62^ 2019	Program impact: increased career development and interest in advanced education and health-related careers for URM students Participant satisfaction: more than 90% program satisfaction	Diversity representation
Brown et al,^40^ 2019	Increased URM representation: in AIDS research Program impact: increased professional and academic development of URM scholars	Academia and research support initiatives
Guevara et al,^61^ 2018	Program impact: increased leadership attainment among scholars Program limitations: did not impact academic productivity, promotions, or retention	Academia and research support initiatives
Spottswood et al,^41^ 2019	Increased URM representation: radiology residency applications and placements	Diversity representation
Germino et al,^42^ 2023	Program impact: enhanced URM mentees’ sense of inclusion in radiation oncology Participant satisfaction: high satisfaction among mentees with mentor attributes	Career advancement and training
Youmans et al,^39^ 2020	Program impact: URM mentor confidence Increased URM representation: increased participation of URM medical students in mentorship activities	Career advancement and training
Odedina et al,^43^ 2022	Program impact: habits of scientific thinking	Academia and research support initiatives
Butler et al,^45^ 2015	Increased URM representation: in surgical subspecialties and academia Program impact: prepared URM residents to excel in their training and transition into practice	Career advancement and training
Murray et al,^46^ 2016	Program impact: strengthened pipeline and retention for underrepresented minority individuals and disadvantaged students to entering nursing Increased URM representation: nursing education and careers	Diversity representation
Pachter et al,^47^ 2015	Program impact: established peer networking and sustainable mentorship connections for long-term career development Increased URM representation: academic pediatrics for residents	Career advancement and training
Llado-Farrulla et al,^48^ 2021	Increased URM representation: in medical residency	Diversity representation
Alli et al,^49^ 2023	Increased awareness: taught inclusion, diversity, antiracism, and equity learning objectives among students Program impact: improvements made to DEI-related curriculum and enhanced student engagement with DEI topics	Growth of pipeline programs
Williams et al,^50^ 2020	Program impact: participants noted improved mentoring skills	Career advancement and training
Metz et al,^51^ 2017	Program impact: increased primary care specialization and practice in medically underserved areas and demonstrated a strong pipeline for diversifying the physician workforce	Career advancement and training
Travers et al,^52^ 2015	Increased URM representation: states with legislation significantly increased enrollment of Hispanic baccalaureate nursing students, with significant outcomes in states like Arkansas, Florida, and California	Diversity representation
Flores et al,^53^ 2021	Program impact: improved mentoring and career trajectories for URM pediatric faculty, increased scholarly output Increased URM representation: in academia society membership	Academia and research support initiatives
Eakin et al,^54^ 2022	Program impact: both in person and remote programs showed a significant increase in participation by underrepresented students, improved student satisfaction, and enhanced career encouragement Participant satisfaction: students in both cohorts viewed program favorably, with remove cohort showing higher engagement	Academia and research support initiatives
Zhou et al,^55^ 2021	Program impact: significant increases conducting research, understanding of physician identity, and sense of preparedness for medical school Increased URM representation: 46 students matriculated to med schools.	Diversity representation
Harris et al,^56^ 2012	Program impact: enhanced cultural competence and career development opportunities for minority participants, contributing to leadership and academic achievements Increased URM representation: significant increase among psychiatry residents	Career advancement and training
Greenway et al,^57^ 2021	Program impact: dental pipeline programs are beneficial for strengthening dental school applications (increased dental school admission testing scores) Increased URM representation: matriculation rates to dental school	Career advancement and training
Degazon et al,^58^ 2012	Program impact: majority of URM nursing students graduated on time and showed enhanced licensure exam success among underrepresented and disadvantaged students. Increased awareness: fostered culturally competent care	Career advancement and training
Wides et al,^59^ 2013	Program impact: significantly increased dental school matriculation rates among disadvantaged and URM students Increased URM representation: enhanced diversity in the dentist workforce and improved access to oral health care for underserved populations	Diversity representation
Vishwanatha et al,^44^ 2019	Increased URM representation: in STEM and biomedical research Program impact: improved leadership, mentoring, and research outcomes at all stages of the academic pipeline	Diversity representation
Brimhall et al,^60^ 2018	Program impact: fostered collaboration, innovation, and psychological safety Increased awareness: enhanced inclusivity and organizational commitment, contributing to job satisfaction and better care outcomes Participant satisfaction: job satisfaction	Diversity representation

Abbreviations: DEI, diversity, equity, and inclusion; GPA, grade point average; STEM, science, technology, engineering, and mathematics; URM, underrepresented minority (refer to Table 1 for detailed definitions).

In academic settings, EDI programs were associated with to increased representation and retention of underrepresented minority individuals in educational institutions and in academia. Programs were associated with a higher number of grant applications from underrepresented minority individuals,^21,27,29,34,40^ and subsequently greater success in securing grant funding,^21,24,25,27,29,34,40,47,53,61^ particularly for research focused on underrepresented minority populations.^40^ Programs were also associated with higher enrollment and representation of underrepresented minority students in advanced medical and scientific education, including Master of Science, Doctor of Philosophy (PhD), Doctor of Medicine (MD), and combined MD-PhD degree programs.^21,28,33^ Several initiatives provided targeted support for clinician-researchers, particularly in pediatrics^47^ and psychiatry,^25,56^ and reported increased publication output and career progression, including the hiring of new underrepresented minority faculty members.^27,28,29,36,61^ Overall, EDI programs seemed to foster inclusive academic environments that supported the professional development of underrepresented minority scholars and educators. Detailed information on these studies and associated initiatives can be found in eTable 2 in Supplement 1.

### Methodological Quality

Using the JBI critical appraisal tool to assess methodological quality, 7 of the 43 studies (16.3%) included in this review were rated as high quality (eTable 3 in Supplement 1).^20,29,41,46,51,56,58^ Of the remaining studies, 20 were rated as moderate (46.5%)^6,27,31,32,33,35,37,38,39,44,47,49,52,53,55,57,59,60,62,63^ and 16 as low (37.2%)^21,22,23,24,25,26,28,30,34,36,40,42,43,45,54^ methodological quality. The variation in quality was mainly a result of inadequate explanation of confounding factors, insufficient follow-up, and subjective outcome assessment criteria.

### Meta-Analysis

**Figure 2.**
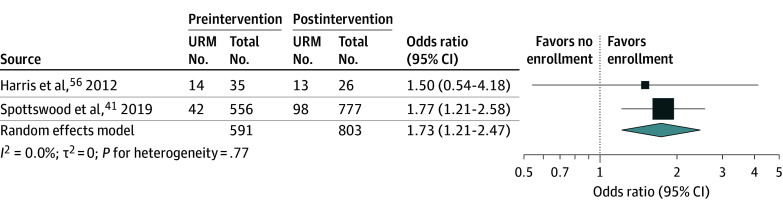
Forest Plot of Analysis Comparing Before-and-After Program Intervention Medical Residency Enrollment Rates of Underrepresented Minority (URM) Populations The URM groups in Harris et al^56^ are detailed in Table 1. For Spottswood et al,^41^ no further details for URM groups were provided.

A random-effects meta-analysis was conducted on data from 2 studies to compare the odds of increased representation of underrepresented minority groups in competitive medical residency enrollment positions preprogram vs postprogram intervention (Figure 2).^41,56^ The pooled OR was 1.73 (95% CI, 1.21-2.47), indicating a statistically significant increase in the odds of underrepresented minority enrollment postintervention. There was no heterogeneity observed (*I*^2^ = 0.0%; τ^2^ = 0; *P* = .77).

## Discussion

In this systematic review composed of 43 articles and more than 15 000 individuals predominantly from the US, who were either enrolled in or supporting EDI-promoting programs, we found a wide range of interventions to be successful in increasing diversity in health care.^20,21,22,23,24,25,26,27,28,29,30,31,32,33,34,35,36,37,38,39,40,41,42,43,44,45,46,47,48,49,50,51,52,53,54,55,56,57,58,59,60,61,62^ Furthermore, the programs assessed appear to be economical, scalable, and often simple in design and implementation. Outcomes assessed across programs included increased underrepresented minority enrollment in medical residency programs, enhanced support and mentorship for standardized health care admission and licensing examinations, increased guidance and encouragement toward careers in academia and research, and higher underrepresented minority enrollment in medical and scientific education. Furthermore, studies reporting participant satisfaction within the programs consistently found it to be high. Our findings suggest that EDI programs in health care institutions are highly successful in improving diversity and representation in their workforce and improving overall satisfaction.

Although prior research has examined EDI initiatives within specific workforce or organizational settings, our review provides a contemporary and more comprehensive synthesis by capturing a wider range of program types and outcomes specifically in health care institutional contexts. For instance, a meta-review by Zhao and colleagues^64^ analyzed 37 studies across multiple workforce sectors (ie, not limited to health care). Their results showed EDI policies involving recruitment, leave, and compensation, as well as workforce accommodations, to be the most studied and used.^64^ These findings partially align with the results of our study, which focused only on health care institutions. In our analysis, minority representation emerged as the most cited and implemented EDI initiatives. However, unlike Zhao and colleagues,^64^ the next most prevalent initiatives in the health care sector were related to career advancement and training, rather than workforce accommodations. This divergence may be explained by the strict educational and training requirements of many health care professionals, such as nurses and physicians. Thus, career advancement and training development may be particularly important in health care, where upstream barriers in education significantly influence representation within the professional workforce.

Next, a different systematic review evaluated the impact of EDI training interventions on race inequalities experienced by health care professionals.^65^ Similar to our findings, this review stated that EDI interventions may improve health care workers’ knowledge and awareness of racial inequalities and cultural competence. Although this review focused more narrowly on health care professionals, it excluded studies that reported on health care students or those that reported solely on patient outcomes. Our review builds on their findings by including student-aged populations, where we believe upstream barriers can be addressed to improve minority representation in the professional workforce. Furthermore, our work expands on this study, assessing EDI initiatives in health care through a meta-analysis of our findings. Our analysis suggests that the odds of enrollment of underrepresented minority individuals in competitive medical residency positions increased by 73% after implementing the mentoring programs.

Program interventions spanned across a range of ages, educational levels, and career development stages. The most common programs targeting underrepresented minority individuals included those implemented for science, technology, engineering, and mathematics (STEM) academia and medical education and residency programs. Less than 20% of studies addressed participant satisfaction with the program; however, those that did consistently reported positive experiences and outcomes. Many programs were simple and cost-effective in design, including volunteer-led mentorship and networking initiatives. However, studies lacked analysis of patient clinical outcomes after program interventions. Clinical outcomes, such as patient length of hospitalization stay, hospitalization readmission rates, and even mortality, provide a clear representation of quantifiable health outcomes for patients.

Previous work has identified improved patient outcomes within diverse health workforces due to greater representation of lived experiences. For instance, a study by Greenwood and colleagues^66^ found that newborn–physician racial concordance was associated with a significant improvement in mortality for Black infants. These results were attributed to factors such as improved communication and trust between parents and their child’s physician. Extending beyond diverse racial workforce representation, a separate study assessed mortality and readmission rates among patients treated by male vs female physicians. Their adjusted analysis found that patients treated by female physicians had a lower 30-day mortality and 30-day readmission rates than those treated by male colleagues.^67^ These differences persist further when assessing female-specific health conditions, such as pregnancy-related concerns. Taken together, these findings suggest that differences in practice patterns across races and sexes exist and may have important clinical implications for patient outcomes. However, these differences need to be studied within the context of EDI programs to enhance their generalizability and reputability.

When considering EDI initiatives in health care, it is important to address multiple domains of equity and inclusion, including gender, sexual orientation, and disability. However, none of the studies in our review reported on outcomes beyond sex, race, and ethnic minority groups, highlighting a need for broader, more inclusive program design and frameworks to promote representation in health care.

Despite the increased attention regarding the promotion of workforce diversity, structural racism and unequal opportunity continue to fuel unequal representation in health care institutions and academia.^9^ Furthermore, recent executive orders targeting the dismantling of EDI programs in the US have had implications worldwide. For instance, British pharmaceutical company GSK removed diversity targets in February 2025 to remain compliant with the law in countries in which they operate, including the US.^68^ Although other pharmaceutical companies, such as AstraZeneca and Novo Nordisk, have remained committed to their EDI initiatives, the pressure from the US, the largest consumer of pharmaceutical goods globally, will continue to exert significant influence over global EDI initiatives across all health care domains.^68,69^

### Strengths and Limitations

This study has a number of strengths. This systematic review is, to our knowledge, the first to assess the impact of EDI programs across various stages of health care careers and health education institutions. Furthermore, our search strategy encompassing a variety of databases yielded a large number of articles for review and analysis. This strategy captured a diverse range of underrepresented minority populations, including variation across both ethnicity and sex. In terms of methodological strengths, the use of 2 independent reviewers for study screening and data extraction helped to minimize the risk of selection bias. Additionally, the application of validated appraisal tools, specifically the JBI checklist for methodological quality, enhanced the interpretations and generalizability of the results.

Despite its strengths, this review also presents some limitations. Program satisfaction was assessed solely from the prescriptive of beneficiaries of the EDI initiatives, without incorporating input from broader interested parties that may have been impacted by these programs. There was also a lack of a standardized definition for terms such as diverse representation, leading to variability in how outcomes were measured and reported, which limits generalizability. Additionally, studies lacked randomization, control groups, and discussions of potential harms, and none evaluated the long-term implications of these programs on patient outcomes. Eight studies were excluded due to lack of full-text availability, and therefore, it is unclear whether their inclusion would have influenced the findings presented. In addition, although we were able to conduct a meta-analysis, the scope of the analysis was narrow as only 2 studies contained data suitable for quantitative synthesis, which limits the generalizability of the findings.

Most EDI programs focused primarily on Black and African American populations, with other underrepresented groups receiving less attention. Although this finding is likely due to the predominance of US-based studies, where this demographic is the second most prevalent underrepresented minority population, it is important to consider when reporting on outcomes.^70^ Consequently, generalizability to other underrepresented minority groups and international contexts is limited. For instance, other forms of underrepresentation, such as sexual orientation, socioeconomic status, family structure, and disability status, remain understudied. Future research should explore a broader range of underrepresented minority populations to understand how initiatives can address their specific needs and should employ more rigorous and standardized methodological approaches to improve comparability across studies. Although some studies addressed short-term program feasibility, none discussed long-term sustainability. This is an important aspect to ensure continued success in benefiting underrepresented minority groups across health care education and workforce settings.

## Conclusions

In this systematic review and meta-analysis of EDI initiatives in health care institutions, multifaceted interventions were found to promote EDI. These programs can be designed to be simple, economical, and scalable to an institution’s needs. Despite these efforts, current models of professionalism in health care are still perceived as noninclusive toward historically marginalized populations.^12^ Continued effort remains vital to progress toward a more inclusive and equitable health care culture.

## Notes

Supplement 1. **eMethods.** PubMed Search Strategy **eTable 1.** Reasons for Study Exclusion During Full-Text Screening **eTable 2.** Detailed Study Descriptions and Outcomes **eTable 3.** JBI Critical Appraisal Results for Quasi-Experimental Studies

Supplement 2.
Data Sharing Statement
