# Supplementary material for: Equity, Diversity, and Inclusion Programs in Health Care Institutions: A Systematic Review and Meta-Analysis
Source: JAMA Netw Open. 2026 Feb 4;9(2):e2555896. doi: 10.1001/jamanetworkopen.2025.55896 (PMC12873802; doi:10.1001/jamanetworkopen.2025.55896)
Supplement: Supplement 1. — eMethods. PubMed Search Strategy eTable 1. Reasons for Study Exclusion During Full-Text Screening eTable 2. Detailed Study Descriptions and Outcomes eTable 3. JBI Critical Appraisal Results for Quasi-Experimental Studies [file jamanetwopen-e2555896-s001.pdf]

Supplemental Online Content

Fremont D, Buh A, Hoar-Stephens C, et al. Equity, diversity, and inclusion programs in health care institutions. *JAMA Netw Open*. Published online February 4, 2026. doi:10.1001/jamanetworkopen.2025.55896

eMethods. PubMed Search Strategy

eTable 1. Reasons for Study Exclusion During Full-Text Screening

eTable 2. Detailed Study Descriptions and Outcomes

eTable 3. JBI Critical Appraisal Results for Quasi-Experimental Studies

This supplemental material has been provided by the authors to give readers additional information about their work.

© 2026 Fremont D et al. *JAMA Netw Open.*

eMethods. PubMed search strategy

("Health Care Facilities, Manpower, and Services"[Mesh] OR "healthcare institution" OR hospital OR "health clinic" OR "nursing home" OR "university" OR "faculty"[Title/Abstract]) AND ("Diversity, Equity, Inclusion"[Mesh] OR "DEI program" OR "DEI initiative" OR "DEI efforts" OR "diversity, inclusion, equity" OR "minority groups" OR "anti-racism"[Title/Abstract]) AND (“Program Evaluation"[Mesh] OR "employee retention" OR "employee satisfaction" OR "workforce diversity" OR "program evaluation" [Title/Abstract])

© 2026 Fremont D et al. *JAMA Netw Open.*

eTable 1. Reasons for Study Exclusion During Full-Text Screening

Study (First author, year, title)	Reason for Exclusion
Abernethy 1999: A mentoring program for underrepresented-minority students at the University of Rochester School of Medicine.	Wrong outcomes
Abraham 2023: An Upstream Reparative Justice Framework for Improving Diversity in Radiology.	Wrong study design
Acosta 2006: Meeting the needs of regional minority groups: the University of Washington's programs to increase the American Indian and Alaskan native physician workforce.	Wrong study design
Agawu 2019: The Influence of Gender and Underrepresented Minority Status on Medical Student Ranking of Residency Programs.	Wrong study design
Aguwa 2023: Diversity, equity and inclusion in ophthalmology.	Wrong study design
Aisen 2003: Diversity-Building Research Training Program	Wrong study design
Andersen 2009: Recruitment and retention of underrepresented minority and low-income dental students: effects of the Pipeline program.	Wrong study design
Andriole 2010: Variables associated with full-time faculty appointment among contemporary U.S. Medical school graduates: implications for academic medicine workforce diversity.	Wrong study design
Antunez 2003: A multidisciplinary, culturally diverse approach to training health professions students.	Wrong study design
Atchison 2009: The Pipeline program at the University of Washington School of Dentistry.	Wrong study design
Atchison 2009: The Pipeline program at the University of Illinois at Chicago College of Dentistry.	No full text access
Atchison 2009: Community-based clinical dental education: effects of the Pipeline program.	Wrong study design
Aysola 2023: Promoting Access to Medical School and Physician Workforce Diversity.	Wrong study design
Balanay 2022: Racial and Gender Diversity Among Students and Faculty in EHAC-Accredited Environmental Health Sciences Programs: Trend Analysis from 2009 to 2021	Wrong study design
Balicer 2011: Reducing health disparities: strategy planning and implementation in Israel's largest health care organization	Wrong study design
Baradaran 2006: A controlled trial of the effectiveness of a diabetes education programme in a multi-ethnic community in Glasgow [ISRCTN28317455].	Wrong study design
Barfield 2012: Differences between African American and Caucasian students on enrollment influences and barriers in kinesiology-based allied health education programs.	Wrong study design
Barlow 2004: Making a Difference for Minorities: Evaluation of an Educational Enrichment Program.	Wrong study design
BascoWTJr 1999: Relationship between primary care practices in medical school admission and the matriculation of underrepresented-minority and female applicants	Wrong study design
Bekaert 2000: Minority integration in rural healthcare provision: an example of good practice.	Wrong Outcomes
Bell 1999: Interventions to improve uptake of breast screening in inner city Cardiff general practices with ethnic minority lists.	Wrong study design
Bennett 2021: Nursing's leaky pipeline: Barriers to a diverse nursing workforce.	Wrong study design
Bheenuck 2007: Race equality education: Implications of an audit of student learning	Wrong study design
BignallONR2nd 2021: Stony the road we trod: towards racial justice in kidney care.	Wrong study design
Blakely 2003: Blueprint for establishing an effective Postbaccalaureate medical school pre-entry program for educationally disadvantaged students.	Wrong study design
Boatright 2023: Strategies and Best Practices to Improve Diversity, Equity, and Inclusion Among US Graduate Medical Education Programs.	Wrong study design
Boyd 2023: Illuminating the path towards inclusivity: strategies to improve workforce diversity in neonatal-perinatal medicine.	Wrong study design
Brown 2007: The road to excellence for minority graduate students.	Wrong Outcomes
Brown 2005: Performance evaluation for diversity programs.	Wrong Study Design
Brown 1991: The Duke Minority Medical Student Summer Fellowship: one program's attempt to attract minority residents.	Wrong Outcomes
Brown 2019: Increasing Diversity and Capacity in HIV Behavioral and Social Science Research: Reflections and Recommendations From the Inaugural Cohort of the Mid-Atlantic CFAR Consortium (MACC) Scholars Program.	Wrong Outcomes
Brown 2005: An oral health education program for Latino immigrant parents.	Wrong Outcomes
Brown 2022: Reach, acceptability, and perceived success of a telehealth diabetes prevention program among racially and ethnically diverse patients with gestational diabetes: the GEM cluster-randomized trial.	Wrong study design
Bush 2007: Park-based obesity intervention program for inner-city minority children.	Wrong Outcomes
Byars-Winston 2011: Integrating theory and practice to increase scientific workforce diversity: a framework for career development in graduate research training	Wrong study design
Canner 2017: Enhancing Diversity in Biomedical Data Science.	Wrong study design
Cantor 1998: Effect of an intensive educational program for minority college students and recent graduates on the probability of acceptance to medical school.	Wrong study design

© 2026 Fremont D et al. *JAMA Netw Open.*

Study (First author, year, title)	Reason for Exclusion
Chakravarty 2020: Did the Medicare Prescription Drug Program Lead to New Racial and Ethnic Disparities? Examining Long-term Changes in Prescription Drug Access among Minority Populations.	Wrong study design
Chicca 2020: Fostering Inclusive Clinical Learning Environments Using a Psychological Safety Lens.	Wrong study design
Childs 2016: Improving workforce diversity in minority and majority institutions.	Wrong study design
Conlon 2015: Development and Evaluation of the Curriculum for BOLD (Bronx Oncology Living Daily) Healthy Living: a Diabetes Prevention and Control Program for Underserved Cancer Survivors.	Wrong study design
Covington 2010: Developing a community-based stroke prevention intervention course in minority communities: the DC Angels Project.	Wrong Outcomes
Crowe 2020: Females and Minority Racial/Ethnic Groups Remain Underrepresented in Emergency Medical Services: A Ten-Year Assessment, 2008-2017.	Wrong study design
D'Agostino 2018: Impact of change in neighborhood racial/ethnic segregation on cardiovascular health in minority youth attending a park-based afterschool program.	Wrong Outcomes
Daley 2006: Improving the retention of underrepresented minority faculty in academic medicine.	Wrong Outcomes
Drouin 2006: Medical students as teachers and role models for their future colleagues	Wrong study design
Drouin 2003: Training Medical Students to Communicate with a Linguistic Minority Group	Wrong Outcomes
D'Souza 2022: The effectiveness of a diversity and inclusion intervention in higher education in the context of COVID-19.	Wrong study design
Enders 2022: Changing the face of academic medicine: an equity action plan for institutions.	Wrong study design
Escallier 2009: Process and outcomes evaluation of retention strategies within a nursing workforce diversity project.	Wrong study design
Eubanks 1990: Workforce diversity in health care: managing the melting pot.	Wrong study design
Fernandez-Repollet 2018: Effects of summer internship and follow-up distance mentoring programs on middle and high school student perceptions and interest in health careers.	Wrong study design
Findler 2007: The challenge of workforce management in a global society: modeling the relationship between diversity, inclusion, organizational culture, and employee well-being, job satisfaction and organizational commitment.	Wrong study design
Fleming 2012: Enhancing minority student retention and academic performance: What we can learn from program evaluations.	Wrong study design
Fleming 2005: Increasing minority representation in the health professions	Wrong study design
Formicola 2009: The Dental Pipeline program's impact on access disparities and student diversity.	Wrong study design
Freeman 1998: Training family medicine faculty to teach in underserved settings.	Wrong study design
Friedman 2007: Enhancing the diversity of the pediatrician workforce.	Wrong study design
Gao 2023: Equity for Sexual and Gender Diverse Persons in Medicine and Dermatology.	Wrong study design
Garcia 2021: Diverse students collaborating to address social determinants of health using listening sessions.	Wrong study design
Gardner 2005: A successful Minority Retention Project	Wrong study design
Gasnier 2022: Establishing a benchmark of diversity, equity, inclusion and workforce engagement in radiation oncology in Europe - An ESTRO collaborative project.	Wrong study design
Geiger 2023: Academic libraries and DEI initiatives: A quantitative study of employee satisfaction	Wrong study design
Geller 2017: Increasing racially and ethnically underrepresented women in medical school through an innovative program.	Wrong study design
Giles 2009: Application of the interactional model of cultural diversity to identify diversity climate factors associated with organizational effectiveness in accredited U.S. physical therapist education programs.	Wrong study design
Gonzalez 1994: Faculty mentors for minority undergraduate students.	No full text access
Graham 2018: Outreach strategies to recruit low-income African American men to participate in health promotion programs and research: Lessons from the Men of Color Health Awareness (MOCHA) project.	Wrong study design
Gravely 2004: Enrichment and recruitment programs at dental schools: impact on enrollment of underrepresented minority students.	Wrong study design
Grumbach 2006: Effectiveness of University of California postbaccalaureate premedical programs in increasing medical school matriculation for minority and disadvantaged students.	Wrong study design
Guerrero 2022: Workforce Diversity and disparities in wait time and retention among opioid treatment programs.	Wrong Outcomes
Haggins 2018: Value of Near-Peer Mentorship from Protégé and Mentor Perspectives: A Strategy to Increase Physician Workforce Diversity.	Wrong Outcomes
Hammond 2022: "Raising the curtain on the equality theatre": a study of recruitment to first healthcare job post-qualification in the UK National Health Service.	Wrong study design
Harris 2022: Advancing Equity in Academic Medicine Through Holistic Review for Faculty Recruitment and Retention.	Wrong study design
Hewlett 2009: The Pipeline program at the University of North Carolina at Chapel Hill School of Dentistry.	Wrong study design
HigginsMCSS 2023: Workforce Diversity of Interventional Radiology Physicians in North America: Reflections on the U.S. Experience.	Wrong study design
Hill 2008: Collaborative co-op as a means to expand BSN enrollment & diversity.	Wrong study design

© 2026 Fremont D et al. *JAMA Netw Open.*

Study (First author, year, title)	Reason for Exclusion
Hill 2023: National Institutes of Health Diversity Supplement Awards by Medical School.	Wrong study design
Holdren 2022: A Novel Narrative Medicine Approach to DEI Training for Medical School Faculty.	Wrong study design
Howe 2001: An investigation of the diversity sensitivity training program: What is the impact of participation?	Wrong study design
Hudson 2020: Workforce Diversity Begins With Program Diversity	Wrong study design
Hussain 2020: Workforce diversity, diversity training and ethnic minorities: The case of the UK National Health Service.	Wrong study design
Hwang 2017: Gender and Ethnic Diversity in Academic PM&R Faculty: National Trend Analysis of Two Decades.	Wrong study design
Janes 1998: An innovative approach for affirming cultural diversity among baccalaureate nursing students and faculty.	Wrong study design
Jeffe 2014: The emerging physician-scientist workforce: Demographic, experiential, and attitudinal predictors of MD-PhD program enrollment.	Wrong study design
Jeske 2022: Beyond inclusion: Enacting team equity in precision medicine research.	Wrong study design
Johnson 2007: Mentoring disadvantaged nursing students through technical writing workshops.	Wrong study design
JohnsonRowsey 2013: Careers Beyond the Bedside: one approach to develop the ethnic minority nursing faculty pool.	Wrong Outcomes
Jones 2023: Casting a Wider Net: Increasing Diversity, Equity, and Inclusive Excellence in Faculty Searches.	No full text access
Jones 2017: Effectiveness of Nursing Student-led HIV Prevention Education for Minority College Students: The SALSA Project.	Wrong Outcomes
Judd 2007: Imi Ho'ola: an educational model for disadvantaged students at the University of Hawai"i School of Medicine.	Wrong study design
July 1998: The Mentoring Program at Fayetteville State University: a key to success for minority students.	No full text aceess
Kaplan 2006: The role of faith-based institutions in addressing health disparities: a case study of an initiative in the southwest Bronx.	Wrong study design
Kuthy 2009: Assessment of the Dental Pipeline program from the external reviewers and National Program Office.	Wrong study design
Leviton 2009: Foreword. Evaluating the Dental Pipeline program: recruiting minorities and promoting community-based dental education.	No full text access
Lillie-Blanton 2008: Reducing racial, ethnic, and socioeconomic disparities in health care: opportunities in national health reform.	Wrong study design
Majumdar 2004: Effects of cultural sensitivity training on health care provider attitudes and patient outcomes.	Wrong study design
Mason 2020: Insights Into Addressing Structural Barriers and Building Specialty Diversity Through a Successful Pipeline Pathway Program.	Wrong study design
McClellandS3rd 2022: The giant triplets impeding black academic physician workforce diversity.	Wrong study design
McGee 2016: "Biomedical Workforce Diversity: The Context for Mentoring to Develop Talents and Foster Success Within the 'Pipeline'".	Wrong study design
Mensah 2022: Profiles in Wisdom: A Survey of Leading Psychiatrists to Inform the Diversity of the Future Psychiatric Workforce.	Wrong study design
Mittman 2011: Forming state collaborations to diversify the nation's health workforce: The experience of the Sullivan Alliance to Transform the Health Professions.	Wrong study design
Muller 1994: Managing diversity in health services organizations.	Wrong study design
Nation 2020: Narrated Dissection Videos and Peer-Mentoring to Enhance Anatomy Performance of Underrepresented Minority Students in Physical Therapy Education.	Wrong study design
Newman 2019: Eliminating Institutional Barriers to Career Advancement for Diverse Faculty in Academic Surgery.	Wrong study design
Nguyen 2022: Building Faculty Educator Skills In Equity And Anti-Racism: A Realist Program Evaluation	No full text access
Nkansah 2009: Fostering and managing diversity in schools of pharmacy.	Wrong study design
Noone 2007: An organized pre-entry pathway to prepare a diverse nursing workforce.	Wrong Outcomes
Norris 2007: Partnering with community-based organizations: an academic institution's evolving perspective.	Wrong study design
Oates 2023: Using an Annual Diversity, Equity and Inclusion Dashboard to Accelerate Change in Academic Radiology Departments.	Wrong study design
Odedina 2019: Increasing the Representation of Minority Students in the Biomedical Workforce: the ReTOOL Program.	Wrong Outcomes
Ofili 2021: The Research Centers in Minority Institutions (RCMI) Consortium: A Blueprint for Inclusive Excellence.	Wrong study design
Ogunyemi 2022: United States Medical School Academic Faculty Workforce Diversity, Institutional Characteristics, and Geographical Distributions From 2014-2018.	Wrong study design
Ogunyemi 2012: Workforce diversity at the Lagos Business School, Pan-African University, Nigeria.	Wrong study design
Omofoye 2023: The Emerging Diverse Radiology Workplace: Case Studies on the Importance of Inclusion in Radiology Training Programs.	Wrong study design
Padula 2002: Linking minority and disadvantaged high school students with health professions training: A win-win situation for students and older adults.	Wrong study design
Page 2011: Faculty diversity programs in U.S. medical schools and characteristics associated with higher faculty diversity.	Wrong Outcomes
Pasick 2003: Increasing ethnic diversity in cancer control research: description and impact of a model training program.	Wrong study design
Peek 2013: "URM candidates are encouraged to apply": a national study to identify effective strategies to enhance racial and ethnic faculty diversity in academic departments of medicine.	Wrong Outcomes
Peek 2012: A study of national physician organizations' efforts to reduce racial and ethnic health disparities in the United States.	Wrong study design
Petrie 2019: Catering to sex, sexual, and gender diversity: An exploratory study on the effects of LGBTI awareness training on aged care staff in Tasmania, Australia.	Wrong Outcomes

© 2026 Fremont D et al. *JAMA Netw Open.*

Study (First author, year, title)	Reason for Exclusion
Poll-Hunter 2023: Increasing the Representation of Black Men in Medicine by Addressing Systems Factors.	Wrong Outcomes
Poole 2022: Slipping through the cracks: Just how underrepresented are minorities within the dental specialties?	Wrong study design
Popper-Giveon 2014: Workforce ethnic diversity and culturally competent health care: the case of Arab physicians in Israel.	Wrong study design
Porter-Wenzlaff 2008: Responding to increasing RN demand: diversity and retention trends through an accelerated LVN-to-BSN curriculum.	Wrong study design
Printz 2016: Cancer, biomedical science leaders strive to improve workforce diversity.	Wrong study design
Rahal 2022: Diversity, equity, and inclusion in gastroenterology and hepatology: a survey of where we stand.	Wrong study design
Rahal 2022: Diversity, Equity, and Inclusion in Gastroenterology and Hepatology: A Survey of Where We Stand.	Wrong Outcomes
Raphael 2019: The Role of Sponsorship in Achieving Workforce Diversity in Academic Pediatrics.	Wrong study design
Raphael 2023: Faculty Perspectives on Diversity, Equity, and Inclusion: Building a Foundation for Pediatrics	Wrong study design
Reece-Nguyen 2023: Diversity, equity, and inclusion within the Society for Pediatric Anesthesia: A mixed methods assessment.	Wrong study design
Reopell 2023: Community engagement and clinical trial diversity: Navigating barriers and co-designing solutions-A report from the "Health Equity through Diversity" seminar series.	Wrong study design
Rogo 2022: Strategies to Increase Workforce Diversity in Pediatric Infectious Diseases.	Wrong study design
Rust 1998: The Morehouse Faculty Development Program: methods and 3-year outcomes.	Wrong study design
Sanner 2010: The impact of cultural diversity forum on students' openness to diversity.	Wrong study design
Smith 2009: Pipeline programs in the health professions, part 1: preserving diversity and reducing health disparities.	Wrong study design
Sokol 2017: How local health departments work towards health equity.	Wrong study design
Sotto-Santiago 2022: A Framework for Developing Antiracist Medical Educators and Practitioner-Scholars.	Wrong study design
Stewart 2020: Creating a Comprehensive Approach to Exposing Underrepresented Pre-health Professions Students to Clinical Medicine and Health Research.	No full text access
Tekian 2001: The impact of mentoring and advising at-risk underrepresented minority students on medical school performance.	Wrong outcomes
Theile 2011: Cultural competence and the delivery of dental hygiene services: A program evaluation of curriculum effectiveness.	Wrong Outcomes
Thomson 2003: Increasing access to medical education for students from medically underserved communities: one program's success.	Wrong Outcomes
Toia 1997: Ethnic Diversification in Clinical Psychology Graduate Training.	Wrong study design
Toolis 2022: "It's a place to feel like part of the community": Counterspace, inclusion, and empowerment in a drop-in center for homeless and marginalized women.	Wrong study design
Townsel 2000: A challenge for the new millennium: Eliminating health disparities and achieving educational and workforce diversity	Wrong study design
Trent 1997: Teaching urban African American students with learning disabilities in inclusive classrooms: Using study groups to facilitate change	Wrong study design
Tse 2003: A baseline assessment and program evaluation of the diversity initiative services (dis) at a healthcare organization.	Wrong study design
Vermund 2018: Recruitment of Underrepresented Minority Researchers into HIV Prevention Research: The HIV Prevention Trials Network Scholars Program	Wrong Outcomes
Vinson 2015: An exploration of workforce diversity management principles and practices in nursing homes.	Wrong study design
Wang 2022: Racial/Ethnic Representation Among American Board of Family Medicine Certification Candidates from 1970 to 2020.	Wrong study design
Whiteside-Mansell 1997: Evaluation of the teaching enhancements affecting minority students (TEAMS) program.	No full text access
Winer 2023: Diversity, Equity, and Inclusion: Advancing Curricular Development and Recruitment.	Wrong study design
Winkleby 2009: Increasing Diversity in science and health professions: A 21-year longitudinal study documenting college and career success	Wrong study design
Yanagihara 2021: Building a Diverse Workforce and Thinkforce to Reduce Health Disparities.	Wrong study design
Yutrzenka 1999: Four Winds: The evolution of culturally inclusive clinical psychology training for Native Americans.	Wrong study design
Zamora 2009: Afterword to the evaluating the Dental Pipeline Program Report: the California Dental Pipeline Program, Phase II.	Wrong study design

© 2026 Fremont D et al. *JAMA Netw Open.*

eTable 2. Detailed Study Descriptions and Outcomes

Study (first author, year)	Program Type	Program Description	Program Outcomes
Mason et al,[20] 2016	Diversity recruitment and retention	Nth Dimensions and American Academy of Orthopedic surgeon's summer internship program<br>• Aimed to address the low numbers of women and underrepresented minorities in orthopedic surgery	For Women:<br>• Increased odds of applying to orthopedic surgery residency (31% vs 1% among national controls)<br>• Odds Ratio: 43.2; 95% CI: 17.2–99.6; p < 0.001<br>For URMs<br>• Increased odds of applying to orthopaedic surgery (31% vs 3% among national controls)<br>• Odds ratio: 14.5; 95% CI: 7.3–27.5; p < 0.001<br>By Race/Ethnicity:<br>• Black students: 29% applied vs 3% national control (p < 0.001)<br>Latino students: 50% applied vs 3% national control (p < 0.001)
Estape et al,[21] 2018	Educational program	National Institutes of Health Clinical Research Education and Career Development R25 program: Goal to increase the participation of under-represented scientists in the nation's biomedical research enterprise by targeting historically URM institutions<br><br>In this paper, the following programs were described:<br>• Master of Science in Clinical and Translational Research program<br>• Master of Science in Clinical Research program	Increased the number of total grant applications and successful funding from Latino or Hispanic scholars and Black or African American scholars<br>• Master of Science in Clinical and Translational Research program scholars secured 77 grants (19.1 million dollars)<br>• Master of Science in Clinical Research program scholars secured 91 grants (43 million dollars), including 56 pilot grants<br>Increased diversity among researchers<br>• University of Puerto Rico Master of Science: Predominantly Latino females<br>Morehouse School of Medicine: Predominantly Black/African America females
Dios et al,[22] 2014	Diversity mentoring and leadership	Diversity Mentoring Program<br>• Included training workshops for mentors to enhance their diversity mentoring skills, with sessions covering topics like culturally appropriate mentoring and addressing intersectionality	Mentors<br>• 16.7% rated the program as "very successful," 58.3% as "successful"<br>• 41.7% were "very satisfied" with their mentee match, 50% were "satisfied"<br>• 75% rated themselves as "effective" or "very effective" in diversity-related mentoring<br>• 50% desired additional diversity-related training<br>• In terms of mentoring on diversity-related issues: 1 very effective, 8 effective, 3 not very effective<br>Mentees<br>• 36.4% rated the program as "very successful," 54.5% as "successful"<br>• 72.7% were "very satisfied" with their mentor match<br>• 90.9% expressed a desire to continue their relationship with their mentor<br>9.1% discussed diversity issues "very frequently," 36.4% "frequently," 45.5% "occasionally"

© 2026 Fremont D et al. *JAMA Netw Open.*

Study (first author, year)	Program Type	Program Description	Program Outcomes
Inglehart et al,[23] 2014	Diversity recruitment and retention	Ypsilanti High School Recruitment of underrepresented minority and low-income students Through Engagement Program<br>• A two-year pipeline mentoring program designed to recruit URM and low-income high school students into dentistry<br>• Included lectures, hands-on activities, shadowing mentors at clinics, and community outreach projects<br>• The program evaluated participants' interest in dentistry, their experience in mentoring activities, and their understanding of dental profession	Career Goals and College Confidence<br>• Mentees were confident about attending college and interested in dentistry or dental hygiene<br>• By program end, 82% felt confident about going to college ($p < 0.01$ vs. baseline)<br>Program Ratings and Satisfaction<br>• Mentees rated the program high in both years (Year 1: Mean 4.38/5.00, Year 2: 4.00 to 5.00/5.00, $p = 0.01$)<br>• 60% of mentees and 100% of mentors wanted to stay in contact ($p < 0.001$ for mentors)<br>Program Engagement<br>• Mentees' enthusiasm improved significantly over time ("looking forward to next Saturday," $p = 0.03$)<br>Interest in Health Careers<br>• Dentistry: Mean 4.38/5.00<br>Dental hygiene: Mean 3.75/5.00
Blanchard et al,[24] 2019	Academia/ research support initiatives	Network of Minority Health Research Investigators program<br>• Supports underrepresented minorities in biomedical research	Increased grant funding and academic promotion through participation in the Network<br>• Increased confidence in manuscript preparation and management skills<br>Motivators for Participation<br>• Career development and social support were the primary motivators<br>Mentorship and Collaboration<br>• Positive feedback on mentorship<br>• Support with research collaborations and health disparities projects<br>Assistance in navigating academic career pathways
Goldstein et al,[25] 2014	Academia/ research support initiatives	Program for Minority Research Training in Psychiatry<br>• Research Fellowships: Provides support for medical students and training covering grant writing, academic research skills, responsible conduct of research, and opportunities to present findings at scientific meetings	Grant Outcomes<br>• 63 of 99 Program for Minority Research Training Program in Psychiatry fellows secured post-fellowship grants<br>Research Completion and Dissemination<br>• 90% completed their fellowship research project, with over 70% publishing their results<br>• 79.6% presented their findings via poster session, and 79.6% gave oral presentations (e.g., scientific meetings, grand rounds, panels)<br>Research Focus<br>• 56.4% conducted research on minority mental health using program funding<br>Career and Research Engagement<br>• 92.5% engaged in research; 50% spent ≥80% of time on research<br>Awards and Appointments<br>• 43% received R01 or K awards (award programs/grants providing salary and/or research support) (vs. 33% Medical Scientist Training Program, 24% s Medical Fellows Program)<br>52% held academic research appointments (vs. 24% Medical Scientist Training Program, 16% s Medical Fellows Program)

© 2026 Fremont D et al. *JAMA Netw Open.*

Study (first author, year)	Program Type	Program Description	Program Outcomes
Rice et al,[27] 2014	Diversity recruitment and retention	Summer Institute Program to Increase Diversity<br>• A mentored career development program for early-career faculty from URM groups and individuals with disabilities in biomedical research<br>• Summer institutes providing intensive training in research methods, grant writing, and career planning), mid-year meetings (progress reviews, networking, and mock grant reviews), mentorship and networking	Research Confidence<br>• Mentees showed increasing confidence in designing research, conducting research, and writing grant applications (18–23% increase in confidence across these areas over the course of the program)<br>Publication Output<br>• At baseline, mentees averaged ~8 total publications each<br>• Across cohorts, there was an overall increase in publication rate from ~0.9 to 1.3 per year<br>• Nearly 100 peer-reviewed manuscripts published during the program<br>Grant Activity: 17/40 grants funded (43% success rate), including R01, R21, and K-series awards (award programs/grants providing salary and/or research support)
Gotian et al,[28] 2017	Diversity mentoring and leadership	Gateways to the Laboratory program<br>• Summer program mirroring the responsibilities of physician–scientists<br>• Included independent research with faculty mentorship, weekly career development workshops and journal clubs, clinical shadowing and presentations (oral, written, poster), multitiered mentoring: each participant paired with a mentor and MD–PhD student mentors.	Advanced Degree Outcomes<br>• 133 alumni received advanced degrees; 54 were pursuing one at the time of reporting<br>○ 106 (80%) were MD, PhD, MD–PhD, or MD/other combinations<br>○ 27 other advanced degrees, 18 of which were health-related<br>Enrolment and Completion Rates<br>• 63% (154 of 245) entered MD–PhD programs (vs. ~20% national average, $p < 0.001$)<br>• 89% completed MD–PhD, MD, or PhD programs<br>Confidence and Representation<br>• Participants reported significantly higher confidence in pursuing MD–PhD training post-program ($p < 0.01$)<br>• Alumni account for ~2% of all African American MD–PhD students nationwide<br>• Alumni represent ~7% of African American MD–PhD students at the sponsoring institution<br>89% of URM participants pursued advanced degrees, significantly higher than national averages for URMs ($p < 0.01$)
Aguila et al,[29] 2010	Academia/ research support initiatives	Minority Institution/Cancer Center Partnership Initiative<br>• Initiative to address cancer health disparities by fostering equal partnerships between Minority-Serving Institutions and Cancer Centers<br>• The program aims at supporting research and training in cancer health disparities through research projects, training programs, faculty recruitment, outreach initiatives	Research Output<br>• Generated 396 peer-reviewed publications<br>• Submitted 298 grant applications: 156 funded (52% success rate)<br>• Cancer health disparities projects increased from 11 to 61<br>Faculty and Institutional Impact<br>• 68 faculty recruited at minority-serving institutions<br>• Minority-serving institutions faculty increased from 3 in 2004 to 65 in 2008<br>• Cancer education curricula developed at minority-serving institutions where none previously existed<br>• Trainees increased from 30 to 1,096<br>Clinical Trial Participation and Outreach<br>• Outreach activities boosted minority patient recruitment to clinical trials<br>At Meharry–Vanderbilt partnership, minority clinical trial enrollment rose from 2.5% to 25%

© 2026 Fremont D et al. *JAMA Netw Open.*

Study (first author, year)	Program Type	Program Description	Program Outcomes
Adhikari et al,[30] 2023	Diversity mentoring and leadership	Black and Minority Ethnic leadership development initiative	Career Progression and Promotions<br>• 20 out of 79 Black and Minority Ethnic nurses and midwifes advanced to higher grades, including senior health board roles<br>• 18 staff promoted<br>• 11 participants changed roles to improve chances of future progression<br>Professional Development and Education<br>• Majority developed deeper understanding of career pathways and grew in expertise and confidence<br>• 8 participants pursued university study courses to enhance promotion potential<br>Ongoing Motivation and Support<br>• Remaining 31 participants felt inspired to seek out promotion opportunities<br>Participants reported increased confidence, improved communication skills, and greater management support
Dillard et al,[31] 2018	Academia/ research support initiatives	Developing a Research Participation Enhancement and Advocacy Training Program for Diverse Seniors, purpose of the program is to increase research awareness in older ethnically/racially minority groups, build advocacy skills and empower older population	Mental and Social Health Outcomes<br>• Both program participants reported greater reductions in depression and improved mental health quality of life, autonomy, and self-efficacy than program 1 only participants<br>• Both groups noted fewer life participation and social issues post-program; however, participants in both programs 1 and 2 had lower physical quality of life<br>Program Satisfaction<br>• High satisfaction across both groups; participants in both programs rated classes more positively and were more likely to continue participation<br>• Participants agreed the program improved their knowledge, activity levels, and self-care<br>Attitudes Toward Research<br>• Participants in both programs showed greater increases in understanding of research and willingness to participate
Corbie et al,[32] 2022	Diversity mentoring and leadership	Clinical Scholars National Leadership Institute<br>• 3-year, intensive leadership program that centers EDI skill development across personal, interpersonal, organizational, and systems domains through its design, competencies, and curriculum	Competency Growth<br>• Participants showed statistically significant gains across all eight Equity Diversity Inclusion domains<br>• Greatest improvement was in Organizational Capacity for Advancing Health Equity (+1.41), followed by Community Engagement (+1.24) and Social Determinants of Health (+1.17).<br>• All changes were statistically significant ($p < 0.01$ or $p < 0.001$)<br>Application and Advocacy<br>• Advocacy emerged as a common theme across all Equity Diversity Inclusion competencies<br>• 140 real-world examples were submitted, including advocacy for marginalized groups, teaching, building partnerships, and personal growth in Equity Diversity Inclusion engagement<br>Impact and Leadership<br>• Participants influenced institutional systems and policies, advanced in their careers, and contributed to policy changes<br>83 leadership activities were tracked, with 37.3% directly addressing Equity Diversity Inclusion issues

© 2026 Fremont D et al. *JAMA Netw Open.*

Study (first author, year)	Program Type	Program Description	Program Outcomes
Goldsmith et al,[26] 2014	Diversity recruitment and retention	Educational Program for Underserved Middle School Students<br>• One-day educational outreach initiative designed for underrepresented minority (URM) seventh-grade students.<br>• Introduces students to health care careers through interactive, hands-on activities. Students participate in pharmacy and physician assistant labs, learn skills like compounding and physical assessments, and engage in informal discussions with faculty and students to foster interest in health sciences.	Understanding of Pharmacy Careers<br>• Pre-program: 30%<br>• Post-program: 89%, Significant increase ($p < 0.001$)<br>Understanding of Physician Assistant Careers<br>• Pre-program: 11%<br>• Post-program: 76%, Significant increase ($p < 0.001$)<br>Interest in Pursuing a Health Care Career<br>• Pre-program: 67%<br>Post-program: 94%, Significant increase ($p = 0.002$)
Maton et al,[33] 2012	Academia/ research support initiatives	The Meyerhoff Scholars Program: strengths-based initiative designed to increase the number of URM students earning STEM PhDs<br>• The program provides comprehensive support, including full scholarships, academic preparation through a Summer Bridge program, mentorship, research opportunities, and a strong community focused on academic excellence.	STEM Graduate Program Entry<br>• Program students were 5.3 times more likely to enter STEM graduate programs than declined students (41.1% vs. 7.8%)<br>• Program students were significantly more likely to enroll in STEM PhD programs (31% vs. 7%)<br>• Program students were more likely to pursue advanced degrees (59% vs. 29%)<br>• Gender did not significantly affect STEM PhD program entry among program students<br>Medical School and Allied Health Program Entry<br>• Program students were less likely to enter medical school (16.8% vs. 29.2%) but equally likely to enter STEM master's or allied health programs<br>Academic Achievement<br>Program students had a higher average GPA (3.45 vs. 3.18) and a higher graduation rate with STEM degrees (89% vs. 55%).
Buchwald et al,[34] 2011	Academia/ research support initiatives	Native Investigator Development Program (career development program): 2-year training initiative aimed at developing American Indian and Alaska Native researchers to address Native health disparities<br>• The program provides intensive mentorship, research training, and professional development, including seminars, statistics and writing instruction, and support for conducting and publishing research projects.	Network Growth: Members collaborated on 106 manuscripts and 83 grant applications.<br>Social Network Analysis Findings<br>• Degree Centrality (direct connections) increased from 0.53 pre-program to 0.83 post-program ($p < 0.01$)<br>• Closeness Centrality (ease of reaching others) improved from 0.47 to 0.76 ($p < 0.01$)<br>• Betweenness Centrality (influence over information flow) increased by 29% post-program ($p = 0.03$)<br>Publications and Grant Activity<br>• Participants collectively published 150 peer-reviewed manuscripts, averaging 4.2 manuscripts per investigator over two years<br>• 68% of participants submitted at least one research grant post-program, and 49% received funding for their grants<br>Mentorship Impact<br>Investigators with more frequent mentor interactions showed greater network connectivity and productivity ($p < 0.05$)

© 2026 Fremont D et al. *JAMA Netw Open.*

Study (first author, year)	Program Type	Program Description	Program Outcomes
Guerrero et al,[35] 2015	Diversity recruitment and retention	Pathways for Students into Health Professions: The program focuses on fostering the development of undergraduate student knowledge, skills, and interest in graduate school training in maternal and child health professions among underrepresented minority (URM) students.	Survey Response and Participant Demographics<br>• 82% response rate with 32 students completing both baseline and 1-year follow-up surveys<br>• Initial reason for applying: Interest in faculty career guidance and mentoring was most important in two of the three years<br>Knowledge Gain<br>• Significant increases in student ratings for knowledge in child health, maternal health, services and programs for children and families, and cultural competency<br>• No significant changes in students' self-rated ability to research maternal child health topics, but significant increases in 11 of 13 maternal and child health topics ($p < 0.05$).<br>Academic and Career Development<br>• Positive changes in ratings for academic and career guidance support, faculty relationships, and interest in pursuing a maternal child health career<br>• Significant increase in ratings for academic and career development ($p < 0.05$) in 6 out of 8 program areas after 1 year<br>Knowledge Scores Over Time<br>Students scored 3 points higher (15%) on average in the knowledge section one year after participating in the program, with the exception of the 2009 cohort
Dossett et al,[36] 2019	Diversity recruitment and retention	Strategies and Tactics for Recruiting to Increase Diversity and Excellence<br>• Included mandatory implicit bias training for all faculty and recruitment committee members<br><br>Diverse Recruitment Committee<br>• Broadened advertising by sharing job posting in traditional and non-traditional venues to attract underrepresented groups<br><br>Modified Rooney Rule<br>• Required at least two diverse candidates in the applicant pool and one interviewed	Demographics of Recruits and Hires<br>• Women: 55% of recruits, 50% of hires<br>• URM's: 15% of recruits, 33% of hires<br>Diversity Improvements<br>• Proportion of URM candidates among hires increased significantly from 6% (baseline) to 33% ($p < 0.05$)<br>• Proportion of women hired also improved, but this change was not statistically significant<br>Participant Feedback<br>• The experience was viewed positively by participants<br>Many recruits felt that the committee and process demonstrated the department's commitment to fostering an inclusive environment

© 2026 Fremont D et al. *JAMA Netw Open.*

Study (first author, year)	Program Type	Program Description	Program Outcomes
Diefenbeck et al,[37] 2021	Diversity recruitment and retention	Nursing Workforce Diversity<br>• The University of Delaware School of Nursing developed a multi-pronged program of recruitment and retention, which utilized evidence-based interventions to address systemic barriers with the overarching goals of increasing recruitment access and retention success for URM/disadvantaged students	Influence of Financial Support<br>• 65% of participants reported that financial support was a significant influence in staying in the nursing program<br>Academic Support<br>• Over time, academic support was primarily considered a "moderate" or "significant" influence on persistence in the nursing major<br>• Most participants found individual or group tutoring (paid by the program) moderately or very helpful in persistence<br>Social/Emotional Support<br>• Social and emotional support was a "moderate" or "significant" influence on persistence, with variations across cohorts<br>• Meetings with the Retention Coordinator were highly valued for providing social, emotional, and cultural support<br>Cultural Competency and Social Engagement<br>• Participants engaged in social/cultural events and organizations, both on and off-campus, with over 90% reporting these activities were helpful in increasing cultural competency<br>Professional Development and Service Learning<br>• 72% of participants rated professional development and service learning/leadership opportunities as having a moderate to significant influence on their persistence in the nursing major<br>Satisfaction with Retention Program<br>• Participants were very satisfied with the Retention Program, with a mean satisfaction rating of 4.85 out of 5<br>Graduation and Retention Rates<br>• 86.2% of participants have graduated or remain enrolled at the university<br>• 68.9% graduated or remain in the nursing program<br>Academic Performance<br>• Participants had cumulative GPAs ranging from 2.3 to 3.95, with a mean GPA of 3.18<br>The GPA of current senior participants (Cohort 3) is 3.36, compared to the 3.4 GPA of senior nursing students in the class of 2020
**Study (first author, year)**	**Program Type**	**Program Description**	**Program Outcomes**

© 2026 Fremont D et al. *JAMA Netw Open.*

Gates et al,[38] 2013	Diversity mentoring and leadership	Bronx-Lebanon Hospital Center Dental Faculty Development Program<br>• A year-long faculty development initiative aimed at enhancing academic career readiness for URM dental faculty and residents<br>• The program included 100 contact hours across four phases, combining didactic sessions, hands-on activities, webinars, and capstone tasks to build skills in teaching, leadership, scholarship, and career development	Knowledge Score Improvement<br>• Pre-instruction mean score: 17.4/36 (48.3%)<br>• Post-instruction mean score: 29.2/36 (81.1%)<br>Changes<br>• Significant changes were found in three specific areas:<br>○ Teaching clinical skills<br>○ Use of standardized evaluations<br>○ Strategies for influencing administrators<br>Confidence Score Improvement<br>• Pretest confidence score: 77.5<br>• Post-test confidence score: 100.2<br>Program Satisfaction<br>• Overall ratings for program implementation and specific sessions were uniformly positive<br>Self-Reported Confidence Increases<br>• Teaching skills: Increased from 3.2 to 4.5 ($p < 0.01$)<br>• Leadership abilities: Increased from 3.1 to 4.4 ($p < 0.01$)<br>Research and scholarship skills: Increased from 3.0 to 4.2 ($p < 0.05$)
Taylor et al,[62] 2019	Diversity recruitment and retention	The Chicago Cancer Health Equity Collaborative Research Fellows Program	Program Satisfaction<br>• Cohort 2: 93% satisfied/highly satisfied<br>• Cohort 3: 100% satisfied/highly satisfied<br>Program Effectiveness<br>• 96% of participants rated the program as highly effective in preparing them for health equity-focused careers<br>Interest in Research Careers<br>• Pre-program: 45% of fellows expressed high interest in pursuing research careers<br>• Post-program: 78% expressed high interest ($p < 0.001$), indicating a significant increase<br>Confidence and Career Interest<br>• Self-reported research confidence:<br>○ Pre-program: 38% rated high confidence.<br>○ Post-program: 75% rated high confidence ($p < 0.01$)<br>• Participants showed increased interest in health-related careers in health equity and research and reported positive changes in career trajectory attitudes<br>Student Outcomes<br>• Community College Transitions: 50% transitioned to 4-year universities.<br>• Medical School: 3 students transitioned to medical school<br>• Graduate-level Health Care Training: 13% transitioned to graduate-level programs<br>• Scholarly Work: 26% presented scholarly work after the fellowship<br>Employment/Internships: 13% obtained positions in Chicago Cancer Health Equity Collaborative -affiliated projects/labs

© 2026 Fremont D et al. *JAMA Netw Open.*

Study (first author, year)	Program Type	Program Description	Program Outcomes
Brown et al,[40] 2019	Academia/ research support initiatives	Mid-Atlantic Center for AIDS Research Consortium Scholars Program<br>• In this program, minority scholars receive mentorship, financial support, professional development, networking opportunities for AIDS research	Program outcomes<br>• URM scholars all experienced supportive mentorship that encouraged both professional and scientific development<br>• Scholars received 4 awards from outside of the Mid-Atlantic Center for AIDS Research Consortium Scholars program<br>• Program established connections with members of the community to enhance their productivity, including community advisory boards and non- Center for AIDS Research affiliated faculty<br>Scholars published five manuscripts, presented at three conferences, and submitted nine grant applications
Guevara et al,[61] 2018	Academia/ research support initiatives	Harold Amos Medical Faculty Development Program<br>• The program provides financial support for academic projects, pairing scholars with national mentors, opportunities to connect with national advisors and peers, training sessions focused on scientific and leadership skills	Grant Success<br>• Scholars received significantly more grants:<br>○ Mean grants: 2.0 for scholars vs. 0.7 for non-scholars ($p < 0.001$)<br>○ Grant funding: $1.46M for scholars vs. $567K for non-scholars ($p = 0.02$)<br>Publications<br>• No significant difference in total publications:<br>○ Mean total publications: 27.2 for scholars vs. 33.0 for non-scholars ($p = 0.47$)<br>○ Mean peer-reviewed publications: 19.5 for scholars vs. 24.4 for non-scholars ($p = 0.39$)<br>Leadership Positions<br>• Scholars were significantly more likely to hold leadership positions:<br>○ 28% of scholars held leadership roles vs. 10% of non-scholars ($p = 0.02$)<br>○ Adjusted Odds Ratio: Scholars had higher odds of attaining leadership roles (AOR 3.9; 95% CI: 1.2–14.0)<br>Promotion and Career Advancement<br>• Scholars were promoted more often, but the difference was not statistically significant:<br>○ 67% of scholars promoted vs. 58% of non-scholars ($p = 0.32$)<br>• A higher percentage of scholars remained in academic medicine, but this difference was also not statistically significant:<br>84% of scholars vs. 75% of non-scholars ($p = 0.21$)
Spottswood et al,[41] 2019	Diversity recruitment and retention	Holistic screening tools for residency application review<br>• A diverse residency recruitment committee<br>• "Second Look Weekend" visits for talented candidates	Increase in URM in medicine Radiology Residency Applicants<br>• The percentage of URM in medicine applicants increased significantly from 7.5% (42 of 556) in the 2012-2013 recruitment year to 12.6% (98 of 777) in the 2017-2018 recruitment year ($P = .001$)<br>Increase in URM in medicine Radiology Residency Representation<br>Representation of URM residents in radiology increased from 0% (0 of 32) in the 2013-2014 academic year to 20% (6 of 30) in the 2018-2019 academic year ($P = .01$)

© 2026 Fremont D et al. *JAMA Netw Open.*

Study (first author, year)	Program Type	Program Description	Program Outcomes
Germino et al,[42] 2023	Diversity mentoring and leadership	Mentorship program incorporating a virtual platform designed by the Association of Residents in Radiation Oncology Equity and Inclusion Subcommittee<br>• Structured to include 6 sessions over 6 months with matched mentor-mentee pairs	Pre-program Satisfaction and Support<br>• Pre-program satisfaction: Only 9.7% (3 mentees) reported satisfaction with current mentorship<br>• Mentorship support for URMs: Only 16% (5 mentees) reported mechanisms or mentorship in place at their program to support URMs<br>Post-program Improvements<br>• High satisfaction among mentees with mentor attributes.<br>• Identified opportunities to improve mentor-mentee engagement and longitudinal program participation<br>Enhanced mentees' sense of inclusion in radiation oncology
Youmans et al,[39] 2020	Diversity mentoring and leadership	Student to Resident Institutional Vehicle for Excellence mentorship program:<br>• Medical school curriculum review sessions, panel discussions, and social events for medical students	Impact of Program on Mentorship<br>• 95% (19 of 20) of respondents agreed that the Student to Resident Institutional Vehicle for Excellence mentorship program made them a better mentor<br>• 90% (18 of 20) reported that they would have appreciated an equivalent program during their medical school training<br>75% (15 of 20) agreed that the program helped them address the challenges of underrepresentation in medicine
Odedina et al,[43] 2022	Academia/ research support initiatives	The Research Training Opportunities for Outstanding Leaders program<br>• Implemented to increase the representation of racial and ethnic minorities in the biomedical workforce	Open-ended responses indicate that program has been instrumental in socializing and acculturating URM participants into the habits of scientific thinking
Vishwanatha et al,[44] 2019	Diversity recruitment and retention	Coordinated Diversity Pipeline Initiative<br>• Participants were exposed to campus tours, hands-on training, and role model seminars<br>• Activities included mentoring, training, summer research, and leadership workshops to increase URM representation in biomedical research	Increased URM Participation<br>• There was an increase in underrepresented minority (URM) participation at all career stages<br>Enhanced Skills<br>• Participants showed improved leadership and research skills<br>Strengthened Institutional Partnerships<br>The program fostered robust institutional partnerships with Hispanic-serving institutions, historically Black colleges and universities, and other minority-serving institutions

© 2026 Fremont D et al. *JAMA Netw Open.*

Study (first author, year)	Program Type	Program Description	Program Outcomes
Butler et al,[45] 2015	Diversity mentoring and leadership	Diverse Surgeons Initiative: A mentorship and training program for URM surgical residents in minimally invasive surgical training and academic surgery.<br>• A 9-month, 3-session training program on minimally invasive surgery fundamentals, skills workshops, mentorship, and exposure to fellowship directors	Success of the Diverse Surgeons Initiative<br>• The initiative has achieved sustained success in preparing underrepresented minority residents to excel in training and transition into practice<br>Professional Achievements<br>• High residency completion rates among participants<br>• Significant attainment of subspecialty fellowships<br>• Notable contributions to peer-reviewed publications<br>Impact on Diversity<br>The program has contributed to increased diversity in academic surgery
Murray et al,[46] 2016	Diversity recruitment and retention	Health Career Clubs and retention initiatives for nursing students<br>• Retention strategies included mentorship, peer support, academic resources, scholarships, and NCLEX prep	Improvements After Health Career Clubs Project<br>• Induction and retention rates improved following the implementation of the Health Career Clubs project<br>Academic Success<br>• Participants showed improved GPAs, increasing from 2.82 to 3.02<br>• Higher NCLEX-RN pass rates were observed among participants<br>Support for Disadvantaged Students<br>The program provided scholarships and proactive academic support for disadvantaged students
Pachter et al,[47] 2015	Diversity mentoring and leadership	The new century scholars' program<br>• Mentorship pairing with senior and junior mentors, travel grants to Pediatric Academic Societies meetings, workshops, and career counselling	Career Outcomes<br>• 63% (38 of 65) participants were in academic careers at the end of the program<br>Impact on URM Representation<br>• The program contributed to increased URM representation in academic pediatrics, with 63% of participants entering academic careers<br>Program Benefits<br>The program enhanced peer support, mentorship access, and professional development among participants
Llado-Farrulla et al,[48] 2021	Diversity recruitment and retention	Representation of physicians underrepresented in medicine in their plastic surgery residency program: A multifaceted recruitment strategy targeting URM students<br>• Included a 4-week visiting clerkship, holistic application reviews, and outreach through the Alliance of Minority Physicians	Increase in URM Representation<br>• The representation of underrepresented-in-medicine (URM) residents in the plastic and reconstructive surgery residency program steadily increased from 0% to 29% with the implementation of a multifaceted approach across a 9 year period<br>Female Representation<br>Female representation was maintained at national averages

© 2026 Fremont D et al. *JAMA Netw Open.*

Study (first author, year)	Program Type	Program Description	Program Outcomes
Alli et al,[49] 2023	Educational program	A 4-day virtual Inclusion, Diversity, Antiracism, and Equity course aimed at addressing gaps in cultural competence and improving DEI-related curriculum	Increase in Learning Objectives<br>• The number of program-taught learning objectives doubled from 43 to 107 (a 149% increase)<br>Improvements in DEI Curriculum<br>• There was a substantial improvement in the DEI-related curriculum<br>Student Outcomes<br>Students demonstrated greater self-awareness, enhanced critical thinking about racism in medicine, and a stronger readiness to address structural inequities
Williams et al,[6] 2020	Diversity mentoring and leadership	Peer Mentor Development Program designed to train and support racially/ethnically diverse junior faculty in mentoring	Improved Mentoring Skills<br>• 53%-86% of skills were rated as "more than before" by participants<br>Sustained Mentor-Mentee Collaborations<br>• Active mentor-mentee collaborations continued post-program, indicating sustained improvement<br>Enhanced Faculty Mentoring Capacity<br>The program contributed to enhanced diversity in faculty mentoring capacity
Metz et al,[51] 2017	Diversity mentoring and leadership	Preparatory pipeline program offering academic enhancement, mentorship, and medical school admissions support for disadvantaged students.<br>• Including science coursework, Medical College Admission Test prep, and professional development	'MED PREP' Program Outcomes<br>• 79% of students completing 'MEDPREP' became practicing physicians<br>Impact on Underrepresented Students<br>• Increased entry and graduation rates in medical school for underrepresented students<br>Physician Diversity and Service Areas<br>The program contributed to greater physician diversity and higher rates of alumni practicing in Medically Underserved Areas and primary care fields
Travers et al,[52] 2015	Diversity recruitment and retention	State legislation focused on minority recruitment into nursing, including funding, reimbursement, workforce enrichment programs, and encouragement strategies	Increased Enrollment of Black Students: These states significantly increased enrollment of Black baccalaureate nursing students<br>• Arkansas: 13.8% to 24.5%<br>• California: 3.3% to 5.4%<br>• Michigan: 8.0% to 10.0%<br>Increased Enrollment of Hispanic Students: These states significantly increased enrollment of Hispanic baccalaureate nursing students<br>• Florida: 11.8% to 15.4%<br>• Texas: 11.2% to 13.9%
Flores et al,[53] 2021	Academia/ research support initiatives	Small research grants, mentoring by nationally renowned senior investigators<br>• Mentoring and networking at an annual breakfast<br>• Annual career-development conference<br>• Monthly mentoring conference calls	Impact of Research in Academic Pediatrics Initiative on Diversity on Scholars<br>• For the 10 Scholars from the first 4 cohorts, the mean score was 4.5 (out of 5) for Research in Academic Pediatrics Initiative on Diversity 's effectiveness in fostering mentoring, developing research skills, and helping Scholars feel more comfortable as URM<br>Scholarly Contributions<br>• 78% of Scholars delivered platform or poster presentations on their projects<br>Impact on Diversity<br>• The program led to significant improvements in diversity within Academic Pediatric Association membership

Study (first author, year)	Program Type	Program Description	Program Outcomes
Eakin et al,[54] 2022	Academia/ research support initiatives	• Accessible short-term research training programs to recruit and retain underrepresented persons	Student Satisfaction and Engagement<br>• Students in both formats viewed the program favourably, with remote cohorts rating some aspects significantly more favourably<br>Higher Engagement and Satisfaction<br>• Remote cohorts showed higher engagement and satisfaction ratings<br>Increased Participation<br>• The remote format led to enhanced participation by students meeting National Institutes of Health disadvantaged criteria
Zhou et al,[55] 2021	Diversity recruitment and retention	• Two consecutive summers of mentored research and enrichment experiences, with the goal of enabling participants' matriculation to Perelman School of Medicine. Penn Access Summer Scholars has been an 8 week on-campus experience	Increased Confidence and Preparedness<br>• Underrepresented students reported significant increases in their self-reported confidence in conducting research, understanding physician identity, and their sense of preparedness for medical school<br>Medical School Matriculation<br>• A total of 46 students matriculated to medical schools
Harris et al,[56] 2012	Diversity mentoring and leadership	The Texas Regional Psychiatry Minority Mentor Network<br>• Mentorship program with quarterly workshops on skills-building, publication, career progression, and cultural competence training	Increased Ethnic Minority Representation<br>• In 2005 and 2006, 14 out of 35 students (40%) entering psychiatry residency were ethnic minorities<br>• After the program's implementation in 2008 and 2009, 13 out of 26 students (50%) were ethnic minorities<br>Enhanced Scholarly Output<br>• The program led to increased scholarly output, including national presentations, publications, and awards<br>Support for URM Participants<br>The program promoted the retention and professional growth of underrepresented minority (URM) participants, addressing barriers to minority representation in psychiatry
Greenway et al,[57] 2021	Diversity mentoring and leadership	Admissions Enhancement Program<br>• A 10-week hybrid summer enrichment program targeting URM and economically disadvantaged students, offering dental admission test reparation, academic support, and mentorship	Effectiveness of Dental Pipeline Programs<br>• Dental pipeline programs strengthened dental school applications, increased Dental Aptitude Test scores, and contributed to greater diversity among dental students<br>• These programs also improved access to care<br>Matriculation and Career Paths<br>• 55% of participants matriculated into dental school, with many pursuing careers in underserved areas<br>Challenges Faced by Scholars<br>Scholars reported facing financial barriers during the application process

© 2026 Fremont D et al. *JAMA Netw Open.*

Study (first author, year)	Program Type	Program Description	Program Outcomes
Degazon et al,[58] 2012	Diversity mentoring and leadership	The Becoming Excellent Students in Transition to Nursing program<br>• Initiatives included academic support (tutoring, study groups), professional counselling, financial aid, and cultural competency training	High Retention and Graduation Rates<br>• 97% of students were retained (84 of 87 students)<br>• 95% of students graduated on time<br>Increased NCLEX-RN Pass Rates<br>• NCLEX-RN first-time pass rate increased to 90%<br>Impact on Health Disparities<br>Graduates contributed to reducing health disparities by serving vulnerable populations in New York City
Wides et al,[59] 2013	Diversity recruitment and retention	Dental Post baccalaureate Program<br>• Provided reapplication assistance to students from economically and/or educationally disadvantaged backgrounds	Impact on Dental Student Diversity<br>• Program graduates are contributing to increased diversity in the dental student population and are helping provide access to care for underserved populations<br>Improved Academic Performance and Acceptance<br>• Participants improved their DAT scores by +1.9 points and achieved a 98% dental school acceptance rate
Brimhall et al,[60] 2018	Diversity recruitment and retention	Workplace Inclusion Framework<br>• Analyzed the relationships between workplace inclusion, innovation, job satisfaction, and perceived quality of care<br>• Development of an inclusive climate through organizational practices, leadership inclusiveness, and trust-building measures	Improved Organizational Environment<br>• The program led to improved psychological safety, trust, and inclusivity within organizational units<br>Positive Outcomes<br>• Positive relationships were observed between inclusivity and key outcomes, including innovation, job satisfaction, and perceived quality of care

** URM = Underrepresented minority; USA = United States of America; STEM = Science, Technology, Engineering, and Mathematics

© 2026 Fremont D et al. *JAMA Netw Open.*

eTable 3. JBI Critical Appraisal Results for Quasi-Experimental Studies

Study	Q1	Q2	Q3	Q4	Q5	Q6	Q7	Q8	Q9	Total (%)
Mason, 2016	1	1	0.5	1	0	1	0	1	1	72.22
Estape, 2018	1	0	0.5	0	0	0.5	0	1	0	33.33
Inglehart, 2014	1	1	0	0	0	0	0	0.5	1	38.89
Blanchard, 2019	1	0.5	0	0	0	0	0	0.5	0.5	27.78
Dios, 2014	1	1	0	0	0	0.5	0	1	0.5	44.44
Goldstein, 2014	1	1	0	0	0	1	0	1	0	44.44
Rice 2014	1	1	0	0	0	1	0	1	1	55.56
Aguila, 2010	1	1	1	0	0	0	1	1	0.5	72.22
Adhikari, 2023	1	1	0	0	0	0	0	0.5	0	27.78
Gotian, 2017	1	1	0	0	0	1	0	0.5	0	38.89
Dillard, 2018	1	1	0.5	0	0	1	0	0.5	0.5	50.00
Corbie, 2022	1	0.5	0	0	0	1	0	1	1	50.00
Maton, 2012	1	1	0.5	0	0	0	1	1	1	61.11
Buchwald, 2011	0	0.5	0.5	0	0	1	0	1	1	44.44
Goldsmith, 2014	0.5	1	0	0	0	0	0	0.5	0.5	27.78
Guerrero, 2015	1	1	0	0	0	1	0	1	0.5	50.00
Dossett, 2019	0.5	1	0.5	0	0	0	0	1	0.5	38.89
Diefenbeck 2021	0.5	1	0.5	0	0	1	0.5	1	0	50.00
Gates, 2013	1	1	1	0	0	0	1	1	1	66.67
Taylor, 2019	1	1	1	0	0	0	1	1	1	66.67
Brown, 2019	0	1	0	0	0	0	0	0.5	0	16.67
Guevara, 2018	0.5	1	0.5	0	0	1	0	1	1	55.56
Spottswood, 2019	1	1	1	0	0	1	1	1	1	77.78
Germino, 2023	0	1	0	0	0	1	0.5	0.5	0.5	38.89
Youmans, 2020	1	1	0.5	0	0	1	0	0.5	0.5	50.00
Odedina, 2022	0.5	1	0	0	0	1	0	0.5	0	33.33
Vishwanatha, 2019	1	1	1	0	0	0	1	1	1	66.67
Butler 2015	1	1	0	0	0	1	0	1	0	44.44
Murray, 2016	1	1	1	0	0	1	1	1	1	77.78
Pachter, 2015	1	1	0.5	0	0	0.5	1	1	1	66.67
Llado-Farrulla 2021	1	0	0	0	0	1	0	1	1	44.44
Brimhall, 2018	0.5	1	1	0	0	0	1	1	1	61.11
Metz, 2017	1	1	1	0	0	1	1	1	1	77.78
Alli, 2023	1	1	0	0	0	0	1	1	1	55.56
Williams, 2020	1	1	1	0	0	0	1	1	1	66.67
Flores, 2021	0.5	1	0	0	0	1	0	1	1	50.00

© 2026 Fremont D et al. *JAMA Netw Open.*

Study	Q1	Q2	Q3	Q4	Q5	Q6	Q7	Q8	Q9	Total (%)
Travers, 2015	0.5	1	1	0	0	0	0	1	1	50.00
Zhou 2021	0.5	1	0	0	0	0	1	1	1	50.00
Harris, 2012	1	1	1	0	0	1	1	1	1	77.78
Greenway, 2021	1	1	1	0	0	0	1	1	1	66.67
Eakin 2022	1	1	0	0	0	0	0	1	1	44.44
Degazon, 2012	1	1	1	0	0	1	1	1	1	77.78
Wides, 2013	1	1	0	0	0	0	1	1	1	55.56

1 = Yes, 0= No, 0.5 = Unclear

Questions

1. Is it clear in the study what is the ‘cause’ and what is the ‘effect’ (i.e. there is no confusion about which variable comes first)?
2. Were the participants included in any comparisons similar?
3. Were the participants included in any comparisons receiving similar treatment/care, other than the exposure or intervention of interest?
4. Was there a control group?
5. Were there multiple measurements of the outcome both pre and post the intervention/exposure?
6. Was follow up complete and if not, were differences between groups in terms of their follow up adequately described and analyzed?
7. Were the outcomes of participants included in any comparisons measured in the same way?
8. Were outcomes measured in a reliable way?
9. Was appropriate statistical analysis used?

© 2026 Fremont D et al. *JAMA Netw Open.*
