# Supplementary material for: Equity, Diversity, and Inclusion Programs in Health Care Institutions: A Systematic Review and Meta-Analysis
Source: JAMA Netw Open. 2026 Feb 4;9(2):e2555896. doi: 10.1001/jamanetworkopen.2025.55896 (PMC12873802; doi:10.1001/jamanetworkopen.2025.55896)
Supplement: Supplement 2. — Data Sharing Statement [file jamanetwopen-e2555896-s002.pdf]

Data Sharing Statement

Fremont. Equity, Diversity, and Inclusion Programs in Health Care Institutions. *JAMA Netw Open*. Published February 04, 2026. doi:10.1001/jamanetworkopen.2025.55896

Data
Data available: No

Additional Information
Explanation for why data not available: n/a
